# Gut microbial signatures and differences in bipolar disorder and schizophrenia of emerging adulthood

**DOI:** 10.1111/cns.14044

**Published:** 2022-12-05

**Authors:** Yi‐huan Chen, Cui‐hong Zhou, Huan Yu, Wen‐jun Wu, Ying‐wei Wang, Ling Liu, Guang‐tao Hu, Bao‐juan Li, Zheng‐wu Peng, Hua‐ning Wang

**Affiliations:** ^1^ Department of Psychiatry Xijing Hospital, Air Force Medical University Xi'an China; ^2^ Department of Ophthalmology Xijing Hospital, Air Force Medical University Xi'an China; ^3^ Institution of Neuroscience Air Force Medical University Xi'an China; ^4^ Department of Psychiatry Southwest Hospital, Army Medical University Chongqing China; ^5^ School of Biomedical Engineering Air Force Medical University Xi'an China

**Keywords:** bipolar disorder, emerging adulthood, gut microbiota, schizophrenia

## Abstract

**Introduction:**

Gut microbial disturbance has been established as potential pathogenesis of mental disorders. However, the signatures and differences regarding patients with schizophrenia (SCH) or bipolar disorder (BD) in emerging adulthood as well as their subtypes have been poorly addressed.

**Methods:**

In the present study, stool samples obtained from 63 emerging adult patients with schizophrenia (SCH), 50 with bipolar disorder (BD), and 40 healthy controls (HC) were analyzed by 16 S rRNA gene sequencing; psychiatric symptoms and psychological, social, and professional functioning were also assessed.

**Results:**

We found that gut microbiota composition was remarkably changed in the patients with SCH and BD. Moreover, the distinct gut microbiome signatures and their potential function in bipolar depression (BP‐D) and SCH with predominantly negative symptoms (SCH‐N) as well as bipolar mania (BP‐M) and SCH with predominantly positive symptoms (SCH‐P) were also observed. Furthermore, we identified diagnostic potential biomarkers that can distinguish BD from HC (38 genera, AUC = 0.961), SCH from HC (32 genera, AUC = 0.962), and BD from Scheme (13 genera, AUC = 0.823). Potential diagnostic biomarkers that can distinguish BD‐D from SCH‐N (16 genera, AUC = 0.969) and BD‐M from SCH‐P (31 genera, AUC = 0.938) were also identified.

**Conclusion:**

This study provides further understanding of abnormal gut microbiome in emerging adulthood patients with SCH and BD and lay the potential foundation for the development of microbe‐based clinical diagnosis for BD and SCH.

## Graphical abstract

The present study observed the signatures and differences of gut microbiome regarding patients with schizophrenia (SCH) or bipolar disorder (BD) in emerging adulthood as well as their subtypes and identified diagnostic potential biomarkers that can distinguish BD from HC (38 microbial panels, AUC = 0.961), SCH from HC (32 microbial panels, AUC = 0.962), and BD from Scheme (13 microbial panels, AUC = 0.823). Potential diagnostic biomarkers that can distinguish BD‐D from SCH‐N (16 microbial panels, AUC = 0.969) and BD‐M from SCH‐P (31 microbial panels, AUC = 0.938) were also identified.

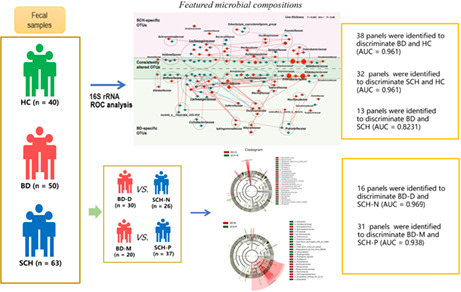

## 1 INTRODUCTION

Bipolar disorder (BD) and Schizophrenia (SCH) are severe mental disorders and are leading causes of morbidity worldwide. BD affects approximately 3% and SCH affects about 1% of the general population worldwide.^1^, ^2^ The diagnosis of BD and SCH still mainly relies on the subjective interpretation of clinical symptoms presented by patients rather than any biological markers and their etiology is not fully understood nowadays. For example, BD is characterized by mood alterations between euthymia, major depression, and mania,^3^ whereas SCH is characterized by hallucinations and delusions.^4^ However, converging evidence suggested similarities between these two disorders such as genetics,^5^, ^6^ clinical phenomenology,^7^, ^8^ and neuroanatomical substrates,^9^, ^10^ and these commonalities are greatest among BD with psychotic features and SCH.^11^, ^12^ Moreover, it is sometimes difficult to clearly distinguish between BD and SCH solely based on phenomenological features, especially symptom performance between bipolar depression (BP‐D) and SCH with predominantly negative symptoms (SCH‐N), as well as bipolar mania (BP‐M) and SCH with predominantly positive symptoms (SCH‐P). Therefore, the investigation of objective biological differences between these subtypes of BD and SCH might be able to provide a basis for the development of new diagnostic methods.

Emerging adulthood is arguably the most unstable period of the lifespan, lasting from age 18 to about age 29 years.^13^ Although emerging adults have reached physical and sexual maturity, they are still learning and maturing and require protection.^14^ It is noteworthy that emerging adults are particularly at‐risk for psychiatric disorders, including bipolar spectrum disorders and schizophrenia.^15^, ^16^ In a given year, over 40% of emerging adults meet the criteria for a psychiatric disorder, a higher rate than for any other adult age group in the U.S.^17^ Moreover, physical and mental comorbidity is prevalent among emerging adults and psychopathology in emerging adulthood undermines adaptation and reduces the likelihood of successful transitions to adulthood.^18^ Furthermore, there is a gap between the need for and the use of mental health services in emerging adults. It was found that 80% of 6–17 year‐olds identified as needing mental health services go untreated^19^ and mental health service utilization drops almost in half, from 34 cases per 1000 among 16–7 year‐olds to 18 per 1000 cases for those 18–19.^20^ Therefore, it is necessary to further study the pathogenesis of emerging adulthood with psychiatric disorders and develop new treatment methods on this basis.

The link between human gut microbiota and neuroendocrine, neuroimmune, and neural and humoral pathways has been widely accepted,^21^, ^22^ and the perturbations in gut microbiota composition in psychiatric disorders have also been concerned.^23^, ^24^ Likewise, the accumulation of knowledge indicates the involvement of gut microbiome in the pathogenesis of BD and SCH.^25^, ^26^, ^27^ Meanwhile, the effects of atypical antipsychotic treatment were associated with measurable differences in gut microbiota in patients with BD or SCH.^28^ Moreover, features of gut microbiota have become established as one of the pivotal tools for biomarker discovery for the diagnosis of SCH and BD.^29^, ^30^ However, thus far, the understanding of the difference in gut microbiota between SCH and BD in emerging adults needs to be further clarified and no studies have directly compared the compositions of gut microbiota between BP‐bpD and SCH‐N, BP‐M, and SCH‐P in emerging adults.

Considering the above, the present study performed a case–control study using 16 S ribosomal RNA (rRNA) gene sequencing and analyses of stool samples obtained from age‐matched emerging adults with SCH (*n* = 63) and BD (*n* = 50) compared with healthy controls (HC, *n* = 40). We sought to identify SCH‐ and BD‐related microbial signatures compared to HC. Next, co‐occurrence analysis based on the relative abundance of altered bacterial operational taxonomic units (OTUs) was performed to construct the key covarying networks in SCH and BD. Finally, we sought to determine the differences in microbial signatures and function abundance between BP‐D (*n* = 30) and SCH‐N (*n* = 26), and between BP‐M (*n* = 20) and SCH‐P (*n* = 37), respectively.

## 2 METHODS

### 2.1 Participant selection

The protocols of this study were reviewed and approved by the Institutional Review Board of the First Affiliated Hospital, School of Air Force Medical University (approval number KY20172048‐F‐2) and was registered with the Chinese Clinical Trial Registry (registration number ChiCTR‐ROC‐17013029). Written informed consent was obtained from all subjects or their guardians. Fifty‐eight patients with BD and 75 patients with SCH were recruited from the Department of Psychiatry at the First Affiliated Hospital of Air Force Medical University, Xi'an, China along with 49 healthy individuals recruited from advertising. According to the inclusion and exclusion criteria, 50 patients with BD, 63 patients with SCH, and 40 healthy controls were included in this study. The patients with BD and SCH, respectively, met the Diagnostic and Statistical Manual of Mental Disorders, 5th Edition's (DSM‐5) criteria for BD and SCH. In addition, the BD‐D patients with a current depressive episode and have a 17‐item Hamilton Depression Scale (HAMD‐17) score ≥ 18 and the BD‐M patients with a current manic episode and have a Young Manic Rating Scale (YMRS) score ≥ 20. Meanwhile, patients with SCH have a total Positive and Negative Syndrome Scale (PANSS) score ≥ 60. SCH‐N was operationally defined as a Baseline score ≥4 on at least 3, or ≥5 on at least 2 negative PANSS subscale items, and PANSS negative subscale score at least 20 and at least 1 point greater than the PANSS positive subscale^31^; SCH‐P were operationally defined as Baseline score ≥4 on at least 3, or ≥5 on at least 2 positive PANSS subscale items, and PANSS positive subscale score of at least 20 and at least 1 point greater than the PANSS negative subscale. Healthy participants were screened through a semi‐structured clinical interview to exclude those with psychiatric or physical illnesses. All participants were between 16 and 25 years old and had not taken prebiotics, probiotics, or antibiotics within 1 month prior to enrollment. The exclusion criteria include digestive diseases such as inflammatory bowel disease and fatty liver; obesity, defined as a body mass index (BMI) ≥ 28.0; hyperglycemia, defined as fasting plasma glucose ≥6.1 mmol/L and/or a diagnosis of/treatment for diabetes; triglyceride levels ≥2.3 mmol/L; hypertension; participants with a severely imbalanced diet, such as high‐fat diet preferences or long‐term vegetarians; pregnancy or lactation; patients with any other psychiatric comorbidities; and receiving any type of psychotropic substances for more than three consecutive days in the 2 weeks before the study begins. The detailed characteristics of these included subjects are shown in Table S1.

Each subject's complete medical history, physical examination, and laboratory test results were recorded. The Mini‐International Neuropsychiatric Interview was used to screen for preexisting psychiatric disorders. Diagnosis of BD and SCH was performed by two psychiatrists. HAMD and YMRS were used to evaluate the severity of depression or mania. The Global Assessment of Function (GAF) scores were used to assess psychological, social, and professional functioning, and the PANSS scores were mainly used to assess the severity of psychiatric symptoms.

### 2.2 Fecal sample collection, DNA extraction, and Illumina MiSeq sequencing

Fecal samples were obtained from all recruited participants and were collected in a sterile plastic cup and stored at −80°C within 0.5 h after collection. A frozen aliquot (200 mg) of each fecal sample was suspended in 250 μl of guanidine thiocyanate, 0.1 M Tris (pH 7.5), and 40 μl of 10% N‐lauroyl sarcosine. Microbial DNA was extracted using the HiPure Stool DNA Kits (Magen, Guangzhou, China) according to the manufacturer's protocols. The quality and concentration of DNA were determined by 1.0% agarose gel electrophoresis and a NanoDrop® ND‐2000 spectrophotometer (Thermo Scientific Inc.) and kept at −80°C prior to further use. The hypervariable region V3‐V4 of the bacterial 16 S rRNA gene was amplified with primer pairs 338F (5′‐ACTCCTACGGGAGGCAGCAG‐3′) and 806R (5′‐GGACTACHVGGGTWTCTAAT‐3′) by an ABI GeneAmp® 9700 PCR thermocycler (ABI). The PCR reaction mixture including 4 μl 5 × Fast Pfu buffer, 2 μl 2.5 mM dNTPs, 0.8 μl each primer (5 μM), 0.4 μl Fast Pfu polymerase, 10 ng of template DNA, and ddH_2_O to a final volume of 20 μl. PCR amplification cycling conditions were as follows: initial denaturation at 95°C for 2 min, followed by 25 cycles of denaturing at 95°C for 30 s, annealing at 55°C for 30 s and extension at 72°C for 30 s, and single extension at 72°C for 5 min, and end at 4°C. All samples were amplified in triplicate. The PCR product was extracted from 2% agarose gel and purified using the AxyPrep DNA Gel Extraction Kit (Axygen Biosciences) according to the manufacturer's instructions and quantified using Quantus™ Fluorometer (Promega). Purified amplicons were pooled in equimolar amounts and paired‐end sequenced on an Illumina NovaSeq PE250 platform (Illumina) according to the standard protocols by Majorbio Bio‐Pharm Technology Co. Ltd.

### 2.3 Data processing

Raw FASTQ files were de‐multiplexed using an in‐house perl script, and then quality‐filtered USEARCH 8.0 with the following criteria: (i) the 400 bp reads were truncated at any site receiving an average quality score of <20 over a 50 bp sliding window, and the truncated reads shorter than 50 bp were discarded, reads containing ambiguous characters were also discarded; (ii) only overlapping sequences longer than 10 bp were assembled according to their overlapped sequence. The maximum mismatch ratio of the overlap region is 0.1. Reads that could not be assembled were discarded; (iii) Samples were distinguished according to the barcode and primers, and the sequence direction was adjusted, exact barcode matching, 2 nucleotide mismatch in primer matching. Then the optimized sequences were clustered into operational taxonomic units (OTUs) using UPARSE 7.1 with 97% sequence similarity level.^32^ The most abundant sequence for each OTU was selected as a representative sequence. To minimize the effects of sequencing depth on alpha and beta diversity measure, the number of 16 S rRNA gene sequences from each sample was rarefied to 20,000, which still yielded an average Good's coverage of 99.09%, respectively. The taxonomy of each OTU representative sequence was analyzed by an RDP Classifier version 2.2 against the 16 S rRNA gene database Silva v138 using a confidence threshold of 0.7.

### 2.4 16S sequence analysis and statistical analysis

Differences in descriptive data, scale scores, and α diversities were assessed with the chi‐squared test for categorical variables. The normal distribution of continuous data was detected by Shapiro–Wilk test. Then the independent‐samples *t*‐test or non‐parametric Mann–Whitney *U* test was used for continuous variables between two subgroups, and the one‐way ANOVA test or Kruskal–Wallis *H* test was used for continuous variables among three groups. Bioinformatic analysis of the gut microbiota was performed using the Omicsmart online platform (http://www.omicsmart.com) and the Majorbio Cloud platform (https://cloud.majorbio.com). Between groups, Venn analysis was performed in the R project VennDiagram package (version 1.6.16), and an upset plot was performed in the R project UpSetR package (version 1.3.3) to identify unique and common OTUs. Sob, Chao1, ACE, Shannon, and Simpson index were calculated in QIIME (version 1.9.1). PD‐whole tree index was calculated in picante (version 1.8.2). Principal coordinates analysis (PCoA) of unweighted unifrac, weighted unifrac, and bray curtis distances were generated in the R project Vegan package (version 2.5.3) and plotted in the R project ggplot2 package (version 2.2.1). A ternary plot of species abundance was plotted using the R ggtern package (version 3.1.0). The PERMANOVA test was used to assess the percentage of variation explained by the treatment along with its statistical significance using the Vegan v2.5‐3 package. The linear discriminant analysis (LDA) effect size (LEfSe) was performed to identify the significantly abundant taxa of bacteria among the different groups (LDA score >2, *p* < 0.05). Then, the co‐occurrence networks deduced from the relative abundance of SCH‐ or BD‐related OTUs were generated using Spearman's correlation coefficient (*r* > 0.4 or < −0.4; *p* < 0.05) and visualized in Cytoscape V.3.7.1. Based on the resulting co‐occurrence network, not only SCH‐ or BD‐specific networks could be identified, but also how these microbes in a particular network correlate with each other could be uncovered. R random Forest package was used to build classification models using profiles of genera with significant differences between the two groups. Using the AUC verification method,^33^ the combination of genera corresponding to the point with the highest AUC value was selected for Receiver operating characteristic (ROC) analysis. SPSS version 19.0 was used for ROC analysis and then GraphPad Prism 8.0 was used to draw ROC curves.^34^ The AUC value has a certain accuracy when it is 0.7 ~ 0.9, and the diagnostic prediction is more accurate when the AUC value is above 0.9. The Overall functional classification of microbial communities was predicted using the PICRUSt2 tool based on the phylogenetic tree. PICRUSt2 uses the Minpath method for functional prediction to determine the minimal pathway for the existence of gene families. Spearman's rank correlation coefficient between environmental factors and species was calculated in the R project heatmap package (version 3.3.1). The *p*‐values were set as two‐tailed with the significance level α = 0.05.

## 3 RESULTS

### 3.1 Clinical and sequencing characteristics of the recruited participants

**FIGURE 1:**
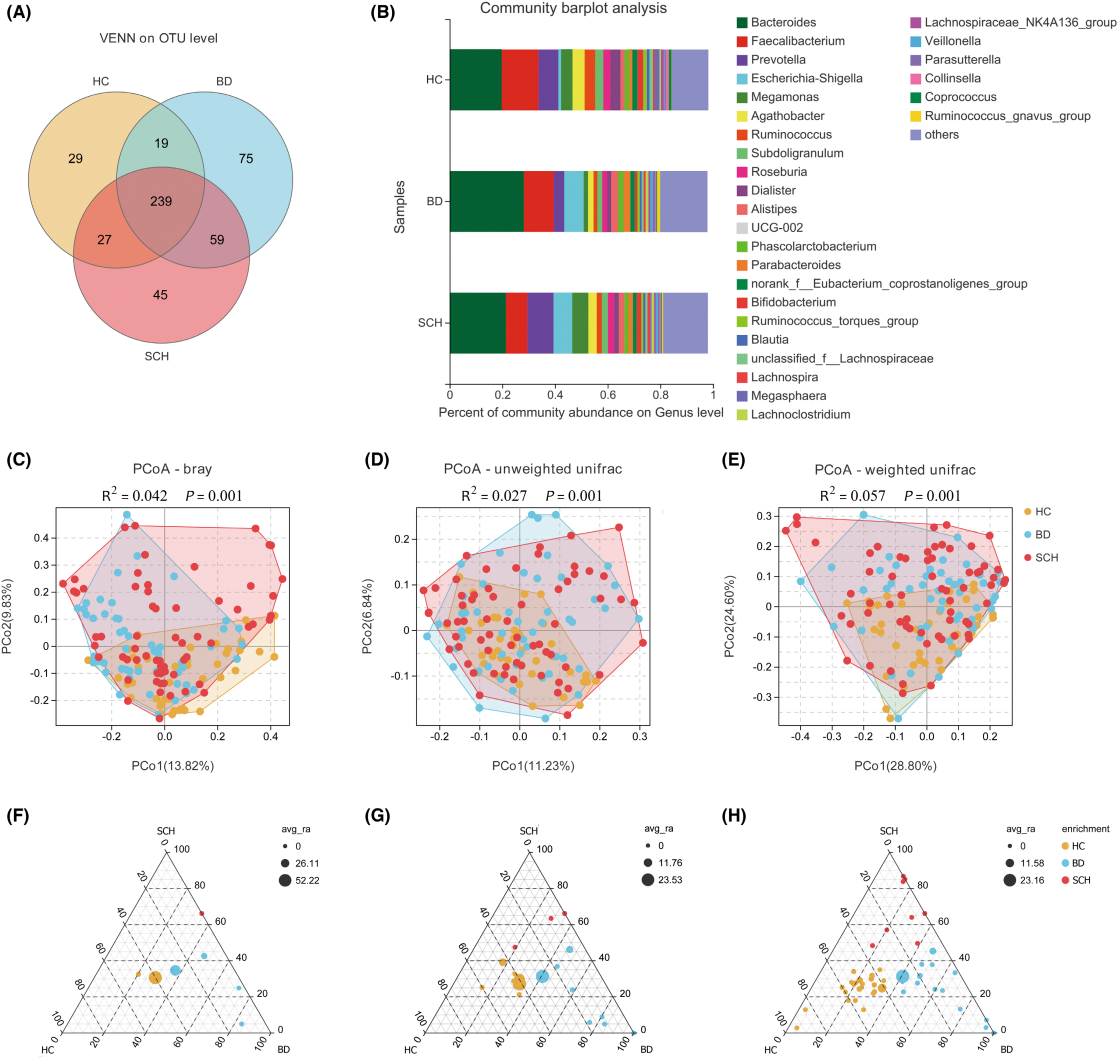
Differential gut microbial characteristics in bipolar disorder (BD; *n* = 50), schizophrenia (SCH; *n* = 63), and healthy controls (HC; *n* = 40). (A) The number of common and unique OTUs among the three groups is displayed by the Venn diagram. (B) The stacked plots are used to visualize the composition of the genus in different groups. Columns of different colors represent different genra, and the length of the column represents the proportion of the genus. (C–E) Principal coordinate analysis showed a clear separation between the three groups at the OTU level using bray curtis, unweighted UniFrac, and weighted UniFrac distance index (PERMANOVA, all *p* < 0.05). Each point in the figure represents a sample. The closer the points on the plane are, the more similar the bacterial colony structure of the sample. The ternary plot was used to display the gut microbiota with significant differences between groups in each group at the phylum (F), family (G), and genus (H) levels. Differential bacteria were represented by different dots, and the size of the dots represents the average abundance. The positions of the dots were composed of the relative abundance ratios of bacteria. Dots marked in yellow were enriched in the HC group, Dots marked in red were enriched in the SCH group, and dots marked in blue were enriched in the BD group.

A total of 40 healthy controls, 50 BD patients, and 63 SCH patients were included in this study after 29 subjects were excluded by the inclusion and exclusion criteria. HC, BD, and SCH subjects were matched for key demographic variables including age (*p* = 0.099), sex (*p* = 0.093), fasting blood sugar (GLU, *p* = 0.190), body mass index (BMI, *p* = 0.065), alcohol intake (*p* = 0.966), and smoking (*p* = 0.226). The detailed characteristics are shown in Table 1. A total of 5,402,452 high‐quality pair‐end reads were filtered from the 5,437,647 raw pair‐end reads. After overlap assembly and tags quality control, 1,389,964 clean tags in healthy control subjects, 1,694,613 clean tags in the BD group, and 2,131,613 clean tags in the SCH group were obtained. These clean tags clustered into 493 qualified Operational Taxonomy Units (OTUs) at 97% sequence similarity for downstream analysis (Figure 1A). At the same time, we found the abundance of *Faecalibacterium*, *Bifidobacterium*, *Agathobacter* was higher in healthy control subjects, whereas Escherichia‐Shigella was more abundant in BD and SCH patients (Figure 1B).

**TABLE 1:** Comparison of clinical characteristics data among the three groups

Demographic and clinical indexes	HC (*n* = 40)	BP (*n* = 50)	SCH (*n* = 63)	*F*/*H*/*χ* ^2^ value	*p* Value
Age (years; M [P25, P75])^b^	21.50 (18.00, 24.75)	20.00 (17.00, 23.00)	21.00 (19.00, 24.00)	*H* = 4.628	0.099
Gender (male/female)^c^	13/27	21/29	34/29	*χ* ^2^ = 4.747	0.093
BMI (kg/m^2^, mean ± SD, range)^a^	22.20 ± 2.83 (16.16–27.45)	21.23 ± 2.59 (16.78–27.76)	20.97 ± 2.52 (16.09–27.47)	*F* = 2.786	0.065
Fasting blood sugar (mmol/L, M [P_25_, P_75_])^b^	4.68 (4.55, 4.97)	4.67 (4.32, 4.84)	4.55 (4.39, 4.79)	*H* = 3.326	0.190
Alcohol intake (Yes/No)^c^	5/34	6/44	7/56	*χ* ^2^ = 0.069	0.966
Smoking (Yes/No)^c^	4/36	7/43	14/49	*χ* ^2^ = 2.971	0.226

Abbreviations: BMI, body mass index; SD, standard deviation.

^a^ One‐way ANOVA.

^b^ Kruskal–Wallis test.

^c^ Chi‐square test.

### 3.2 Diversity and distinct gut microbiome abundance among HC, BD, and SCH and its correlation with clinical characteristics

There were no significant differences in α‐diversity indices, including Observed species (sobs), chao1 and ACE, Simpson and Shannon, and PD‐whole tree among the three groups (Figure S1A–F). However, principal coordinate analysis (PCoA) showed the three groups could be distinguished at the OTU level and permutational multivariate analysis of variance (PERMANOVA) also showed significant differences between the three groups, respectively, by bray curtis (*R* ^2^ = 0.042, *p* = 0.001), unweighted unifrac (*R* ^2^ = 0.027, *p* = 0.001), and weighted unifrac (*R* ^2^ = 0.057, *p* = 0.001) (Figure 1C–E). Moreover, the relative abundance of gut microbiota compositions was compared by Ternary plot (Figure 1F–H). At the phylum level, *Bacteroidetes*, *Proteobacteria*, *Synergistetes*, and *Epsilonbacteraeota* were enriched in the BD group compared with HC or SCH. Meanwhile, *Euryarchaeota* was enriched in the SCH group and *Firmicutes* was more abundant in the HC group. Furthermore, 18 microbiotas at the family level and 48 microbiotas at the genus level were identified to be differentially abundant in HC, BD, or SCH (Details were shown in Table S2). We performed heat map analysis to investigate the association between clinical characteristics and gut microbiota of the genus level (Figure S1G). The abundance of *Alistipes* was enriched in females, whereas *Ruminococcus_gnavus_group*, *Romboutsia*, and *Megamonas* were enriched in male emerging adults. The abundance of *Streptococcus*, *Anaerostipes*, *Bifidobacterium*, and *Ruminococcus_gnavus_group* was negatively correlated with age, whereas *Coprococcus*, *Lachnospira*, and *Alloprevotella* were positively correlated with age. The abundance of *Parabacteroides* and *Alistipes* was negatively correlated with GLU levels. Finally, levels of *Coprococcus*, *NK4A214_group*, *Alloprevotella*, *Clostridia_UCG‐014*, *Lachnospiraceae_NK4A136_group*, and *Eubacterium_eligens_group* were positively correlated with BMI, whereas *Bifidobacterium* and *Ruminococcus_gnavus_group* were negatively correlated with BMI.

### 3.3 Featured microbial compositions in BD or SCH subjects

**FIGURE 2:**
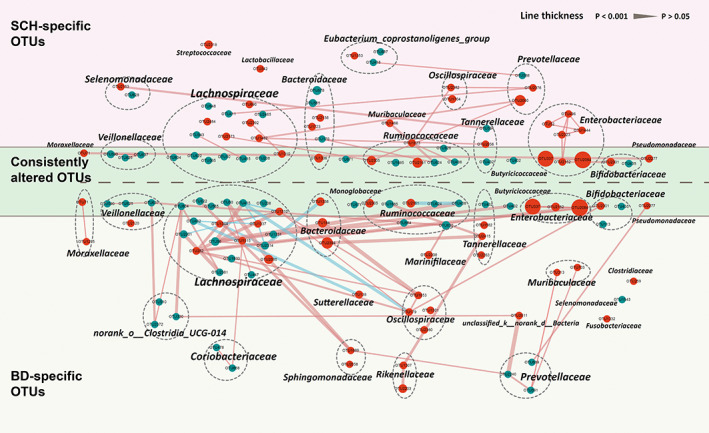
The co‐occurrence network reflects microbial differences in BD and SCH compared with HC. The microbial OTUs changed in SCH or BD was identified by LEfSe and LDA analyses (LDA >2.5). In total, 26 of 108 OTUs were consistently altered in both SCH and BD relative to HC (dark green area), while the majority of OTUs were specific to SCH alone (35/61) (pink area) or BD (47/73) alone (light green area). The size of the node scales with the relative abundance of the OTU. Red dots: increased relative abundance in SCH or BD compared with HC; Green dots: decreased relative abundance in SCH or BD relative to HC. The color of the line represents a positive correlation (Pink) or negative correlation (light blue) according to the Spearman correlation. OTUs annotated to the family level were marked. Nodes of the same family are distributed in the same ellipse. Lines between nodes indicate Spearman's correlation <−0.4 (light green), or >+0.4 (light red); the smaller the *p* value, the thicker the line.

In total, we identified 108 OTUs to be differentially abundant in the BD and SCH groups compared with the HC group (Figure 2). In detail, 26 OTUs were consistently changed in both BD and SCH groups. Accordingly, the majority of altered OTUs were specific to patients with either Scheme (35/61) or BD (47/73). Compared with HC, SCH was characterized by increased OTUs belonging to several families including *Lachnospiraceae* (6 OTUs), *Enterobacteriaceae* (7 OTUs), *Bacteroidaceae* (3 OTUs), and *Ruminococcaceae* (3 OTUs) and decreased OTUs including *Lachnospiraceae* (10 OTUs), *Ruminococcaceae* (4 OTUs), *Bacteroidaceae* (3 OTUs), and *Veillonellaceae* (3 OTUs). BD was characterized by enriched OTUs belonging to the families including *Lachnospiraceae* (6 OTUs), *Oscillospiraceae* (4 OTUs), *Bacteroidaceae* (3 OTUs), and *Enterobacteriaceae* (3 OTUs), and decreased OTUs belonging to the families including *Lachnospiraceae* (14 OTUs), *Ruminococcaceae* (5 OTUs), *Veillonellaceae* (3 OTUs), and *Prevotellaceae* (3 OTUs) (Table S3). Moreover, the statistical covariation among altered OTUs was further analyzed by the co‐occurrence network (Figure 2). The SCH group displayed disturbed covarying OTUs mainly belonging to families including *Lachnospiraceae*, *Prevotellaceae*, and *Ruminococcaceae*. These OTUs were positively covariant with each other. Meanwhile, we found that 7 *Enterobacteriaceae* OTUs (OTU496, 52, 1444, 2323, 337, 2152, and 2084) were positively covaried with one another. In the BD group, the covarying networks constructed by altered OTUs were relatively complex and diverse. The covarying networks generated by *Lachnospiraceae*, *Oscillospiraceae*, and *Bacteroidaceae* were robust but complex, as the OTUs belonging to the three families were both up‐ and downregulated in BD relative to HC and also both positively and negatively correlated with one another. Moreover, OTUs of *Prevotellaceae*, *Enterobacteriaceae*, and other families were positively correlated with one another. Finally, these 108 differential OTUs clustered mainly into several covarying networks comprised of 22 *Lachnospiraceae* OTUs, 4 *Bacteroidaceae* OTUs, 7 *Enterobacteriaceae*, and 8 *Ruminococcaceae* OTUs. Together, these results indicate that SCH and BD share a small proportion of their gut microbial phenotypes but have significantly different microbial signatures.

### 3.4 Clinical characteristics and distinct gut microbiome signatures in BD‐D and SCH‐N

**FIGURE 3:**
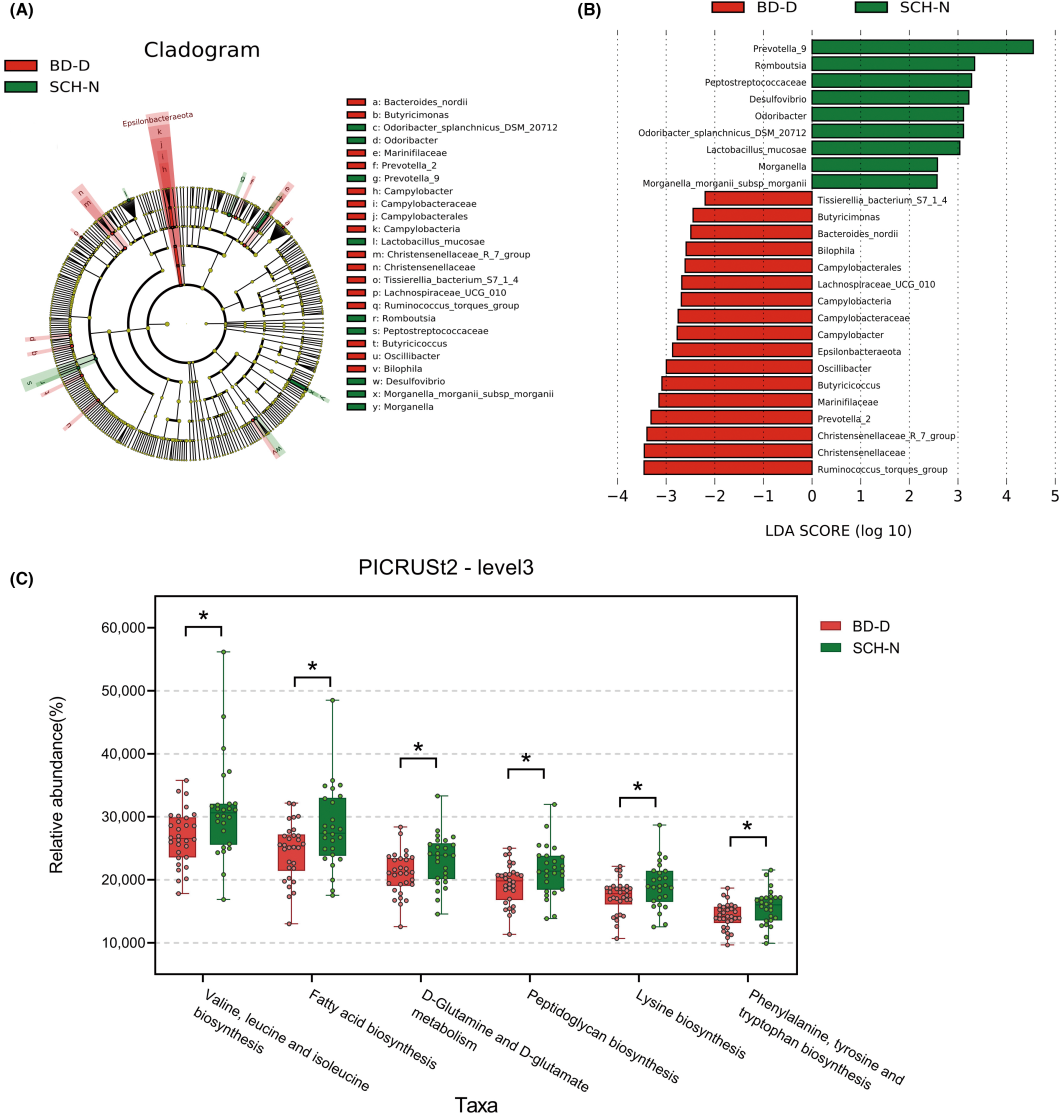
Differences in taxonomic composition and function abundance between the BD‐D group (red) and the SCH‐N group (green). Taxonomic cladogram (A) and LDA scores (B) showed significant bacterial differences between these two groups based on the LEfSe and LDA analyses. Only taxa with an LDA significance threshold >2.0 was presented. Boxplot (C) showed the differences in the functional abundance of important bacterial taxa between subjects with BD‐D and SCH‐N using PICRUSt2 based on the KEGG database (Kruskal–Wallis rank‐sum test), each dot represents the relative abundance of a sample. *p < 0.05. KEGG. Kyoto Encyclopedia of Genes and Genomes.

A total of 30 BD‐D patients and 26 SCH‐N patients were recruited. There were no significant differences in family history and total disease course between these two subgroups. The HAMD‐17 and GAF scores were significantly higher in the BD‐D group compared with the SCH‐N group, whereas PANSS total score and negative score in the SCH‐N group were significantly higher than that of the BD‐D group. About 1,065,539 high‐quality pair‐end reads and 368 OTUs were obtained from BD‐D patients and 939,178 high‐quality pair‐end reads and 326 OTUs were obtained from SCH‐N patients. There is no significant difference between BD‐D and SCH‐N in α‐diversity indices and the fecal microbiotas of the two subgroups could be divided into clusters according to community composition using bray curtis (*R* ^2^ = 0.028, *p* = 0.048) (Table S4). Further, Lefse analysis showed that *Epsilonbacteraeota* at the phylum level, *Christensenellaceae*, *Campylobacteraceae*, and *Marinifilaceae* at family level, *Butyricicoccus*, *Ruminococcus_torques_group*, *Oscillibacter*, *Lachnospiraceae_UCG_010*, *Prevotella_2*, *Campylobacter*, *Butyricimonas*, *Christensenellaceae_R_7_group*, and *Bilophila* at genus level were enriched in BD‐D patients, whereas *Peptostreptococcaceae* at the family level, *Romboutsia*, *Morganella*, *Prevotella_9*, *Desulfovibrio*, and *Odoribacter* at the genus level were enriched in SCH‐N patients (Figure 3A,B). Functional prediction of important bacterial taxa between the BD‐D and SCH‐N was achieved using PICRUSt2. A total of 19 pathways were found to differ in abundance between the two subgroups (*p* < 0.05, Table S5). Eighteen pathways that were mainly belonged to metabolism pathways such as fatty acid biosynthesis, Peptidoglycan biosynthesis, and amino acid‐associated pathways were enriched in the SCH‐N group and only the Wnt signaling pathway was enriched in the BD‐D group (Figure 3C). We also analyzed the associations of gut microbiota with clinical parameters between the BD‐D and SCH‐N groups (Figure S2A). The abundance of *Butyricicoccus*, *Alistipes*, and *Odoribacter* was positively correlated with HAMD scores but negatively correlated with negative scores of PANSS. The abundance of *Romboutsia* was negatively correlated with HAMD scores but positively correlated with negative scores of PANSS.

### 3.5 Clinical characteristics and distinct gut microbiome signatures in BD‐M and SCH‐P

**FIGURE 4:**
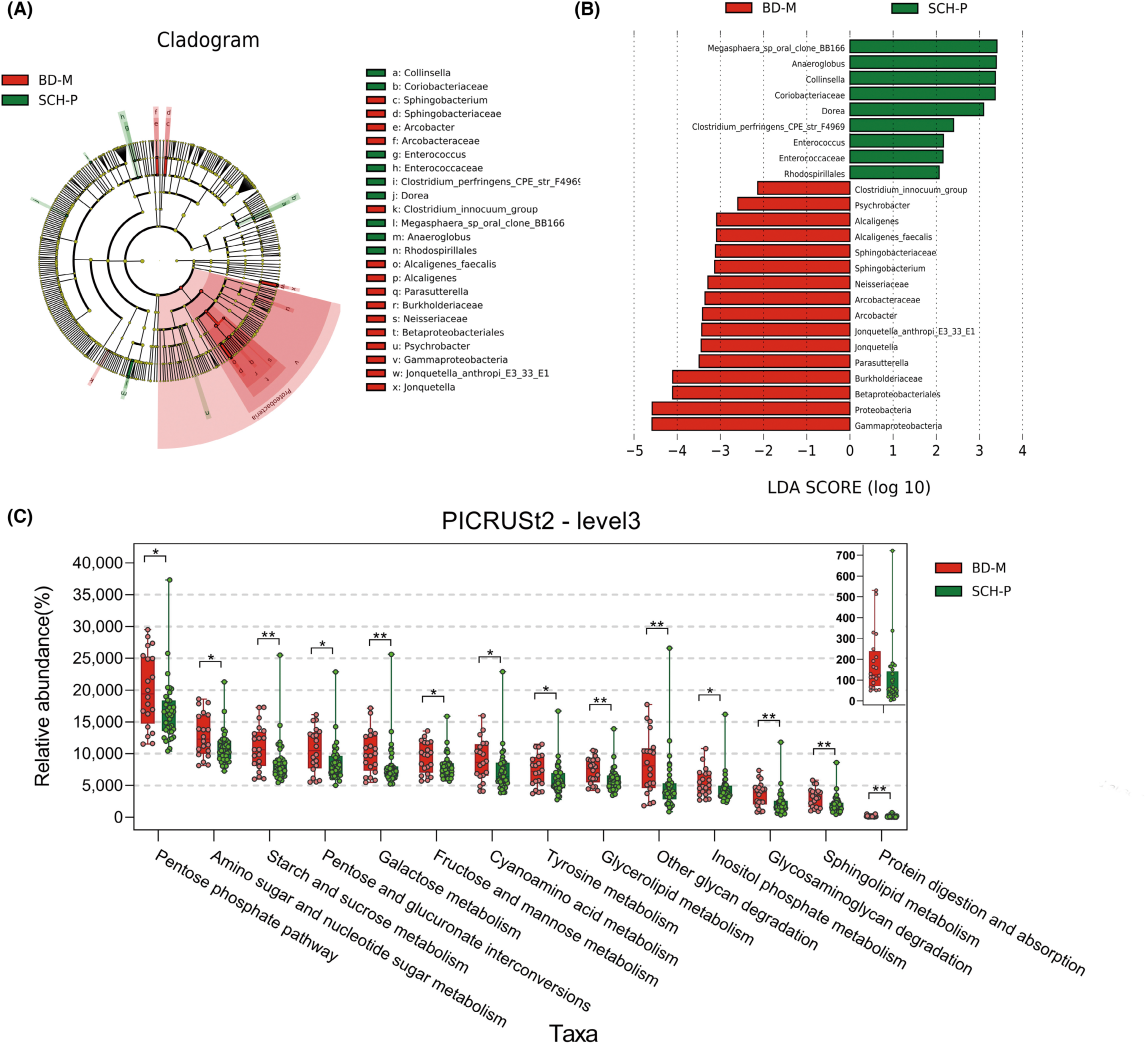
Differences in taxonomic composition and function abundance between the BD‐M group (red) and the SCH‐P group (green). Taxonomic cladogram (A) and LDA scores (B) showed significant bacterial differences between these two groups based on the LEfSe and LDA analyses. Only taxa with an LDA significance threshold >2.0 was presented. Boxplot (C) shows the differences in functional abundance between these two groups using PICRUSt2 based on the KEGG database (Kruskal–Wallis rank‐sum test). each dot represents the relative abundance of a sample. *p < 0.05, **p < 0.01. KEGG, Kyoto Encyclopedia of Genes and Genomes.

There were no significant differences in family history and total disease course between the BD‐M group (*n* = 20) and the SCH‐P group (*n* = 37). In addition, the young mania rating scale (YMRS) and GAF scores were relatively higher in the BD‐M group, whereas PANSS total score and positive score were relatively higher in the SCH‐P group (Table S6). About 694,683 high‐quality pair‐end reads and 668,643 clean tags were obtained from BD‐M patients, and 1287,575 high‐quality pair‐end reads and 1,229,738 clean tags were obtained from SCH‐P patients. These clean tags clustered into 493 qualified OTUs at 97% sequence similarity, of which 339 OTUs were in the BD‐M group and 344 OTUs in the SCH‐P group. There was no significant difference in α‐diversity indices between the BD‐M and SCH‐P groups and the microbiotas could not be divided into clusters according to community composition using bray curtis (*R* ^2^ = 0.025, *p* = 0.112), unweighted UniFrac (*R* ^2^ = 0.019, *p* = 0.353), and weighted Unifrac (*R* ^2^ = 0.016, *p* = 0.440). Lefse analysis showed that *Proteobacteria* phylum, *Arcobacteraceae*, *Neisseriaceae*, *Burkholderiaceae*, and *Sphingobacteriaceae* family, *Jonquetella*, *Clostridium_innocuum_group*, *Parasutterella*, *Arcobacter*, *Sphingobacterium*, and *Alcaligenes* genera were consistently higher in BD‐M patients, while *Coriobacteriaceae* and *Enterococcaceae* family, *Dorea*, *Collinsella*, *Anaeroglobus*, and *Enterococcus* genera were enriched in SCH‐P patients (Figure 4A,B). Functional prediction of important bacterial taxa between BD‐M and SCH‐P groups was also achieved using PICRUSt2. A total of 32 pathways were found to differ in abundance between the two subgroups (*p* < 0.05, Table S5). We observed that the protein digestion and absorption pathway and metabolism pathways were enriched in the BD‐M group, especially carbohydrate, lipid, and amino acid metabolism compared with the SCH‐P group (Figure 4C). The associations of gut microbiota with the clinical parameters between the BD‐M and SCH‐P were also investigated and the abundance of *Coprococcus* and *Ruminococcus* were positively correlated with the positive score and total score of PANSS (Figure S2B).

### 3.6 Potential diagnostic markers of Microbiome

**FIGURE 5:**
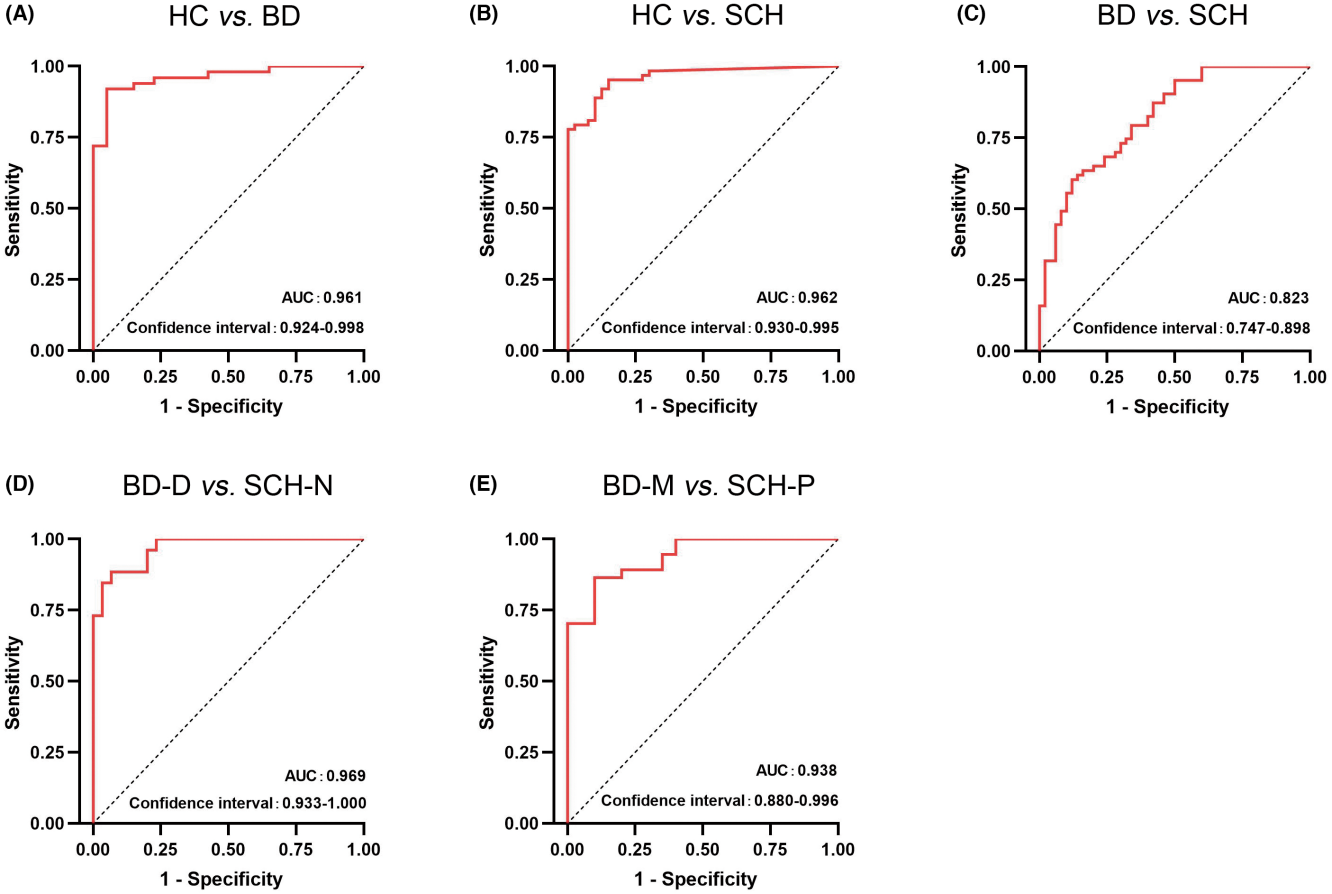
The AUC value of ROC analysis reflects the differential diagnostic potential of microbial markers for discriminating MDD, BD, and HC. (A) HC vs. BD, AUC = 0.961; (B) HC vs. SCH, AUC = 0.962; (C) BD vs. SCH, AUC = 0.823; (E) BD‐D vs SCH‐N, AUC = 0.969; (E) BD‐M vs SCH‐P, AUC = 0.938. *Y*‐axis represents the value of sensitivity; the *X*‐axis shows the value of 1−specificity. The value of AUC in those figures is the area under the corresponding curve. The larger the area under the curve, the higher the diagnostic accuracy.

The general markers were identified from Random Forest classifiers (Figure S3) and are listed in Table S7. The diagnostic potential of microbial markers was assessed by using the area under the ROC curve (AUC). We found that 38 genera could effectively distinguish BD from HC (BD vs. HC, AUC = 0.961, 95% CI: 0.924–0.998, Figure 5A), 32 genera could effectively distinguish SCH from HC (SCH vs. HC, AUC = 0.962, 95% CI: 0.930–0.995, Figure 5B), and 13 genera could effectively distinguish BD from Scheme (BD vs. SCH, AUC = 0.823, 95% CI: 0.747–0.898; Figure 5C). Furthermore, 16 genera could effectively distinguish BD‐D from SCH‐N (BD‐D vs. SCH‐N, AUC = 0.969, 95% CI: 0.933–1, Figure 5D), and 31 genera could effectively distinguish BD‐M from SCH‐P (BD‐M vs. SCH‐P, AUC = 0.938, 95% CI: 0.880–0.996, Figure 5E).

## 4 DISCUSSION

In this study, we characterized the featured microbial compositions and their association with clinical parameters in BD or SCH subjects of emerging adulthood. Meanwhile, the distinct gut microbiome signatures and its potential function in BD‐M and SCH‐P, BD‐D, and SCH‐N were also observed. Moreover, we identified diagnostic potential biomarkers comprising several microbial panels, which may distinguish BD from SCH, and each from HC, as well as BD‐D from SCH‐N and BD‐M from SCH‐P, with high reliability. These results indicated that BD or SCH patients and HC in emerging adulthood as well as the subgroups with similar symptoms could potentially be distinguished by the gut microbiota.

The interaction of the gut microbiota and central nervous system and the influence of gut microbiota on the pathophysiology of mental disorders have been widely reported in recent decades.^35^ The gut microbiota characteristics of patients with BD and SCH have also attracted attention. For instance, the abundance of *Faecalibacterium* was decreased in BD patients compared with healthy subjects and was positively associated with self‐reported symptoms and disease severity,^36^ another work further found that *Bacteroidetes* phylum, *Parabacteroides*, *Bacteroides*, and *Halomonas* genera were greatly enriched in BD patients, while *Firmicutes* phylum, *Roseburia*, *Faecalibacterium*, and *Coprococcus* genera were consistently higher in HCs and 30 microbial markers are identified with moderate effectiveness to distinguish BD and HC.^37^ In terms of SCH, the previous study found that SCH patients had a decreased microbiome α‐diversity index and OTUs mainly belonged to the bacterial taxonomic families such as *Veillonellaceae*, *Prevotellaceae*, and *Bacteroidaceae* were increased whereas bacterial families such as *Lachnospiraceae*, *Ruminococcaceae* and *Enterobacteriaceae* were decreased in patients with SCH compared with HC subjects.^38^ Shen et al.^39^ found that *Proteobacteria*, *Collinsella*, and *Klebsiella* were increased, whereas *Blautia* and *Coprococcus* were decreased in patients with SCH and identified 12 microbiotas as diagnostic factors for distinguishing patients with SCH and HC. Consistent with these results, we found that *Bacteroidetes* phylum and *Bacteroides* genera were enriched in BD and *Firmicutes* phylum, *Faecalibacterium*, and *Coprococcus* genera were enriched in HC. Moreover, we also found that the *Proteobacteria* phylum and *Lachnospiraceae* family, *Blautia*, and *Coprococcus* genera were decreased in patients with SCH. However, we found 48 microbiotas at genus levels were different among HC, BD, and SCH. In detail, 23 genera were enriched in HC, 17 genera were enriched in BD, and 8 genera were enriched in SCH. In addition, the discrepancy with the aforementioned studies, we identified 38 or 32 genera as diagnostic factors for distinguishing patients with BD and HC or SCH and HC. Moreover, there is a controversy over α‐diversity in previous studies,^23^, ^35^ and we did not observe differences in α‐diversity among the three groups. These differences may be related to the age and sex composition of enrolled patients. Of course, it may also be related to inconsistencies in research schemes and statistical methods.

Accumulated literatures suggest that certain characteristics of microbiome may be associated with the severity of mood and psychiatric symptoms, psychiatric medication use, and overall global functioning of SCH and BD.^40^, ^41^ Moreover, the similarities and differences in gut microbiome between major depressive depression (MDD), SCH, and BD have also been reported in a recent systematic review. In detail, higher *Megasphaera* and lower *Roseburia* were common to both SCH and BD compared with MDD. However, higher *Prevotella* and lower *Bacteroides*, *Haemophilus*, and *Streptococcus*, were observed in SCH, whereas higher *Bifidobacterium* and *Oscillibacter* were observed in BD at genus level.^35^ Similarly, the present study also found that *Bacteroides* and *Oscillibacter* were enriched in BD. However, there is no significant between SCH and BD in *Haemophilus*, *Streptococcus*, and *Bifidobacterium*, and higher *Prevotella* were also observed in BD which was inconsistent with previous research results. To further compare the difference and similarities between the two diseases, we utilized a co‐occurrence network to reflect the microbial differences in BD, SCH compared with HC according to the recent study.^42^ On the one hand, 26 of 108 OTUs were consistently altered in both MDD and BD relative to HC, which mainly belongs to *Lachnospiraceae* (7 OTUs), *Ruminococcaceae* (5 OTUs), *Enterobacteriaceae* (3 OTUs), and *Veillonellaceae* (3 OTUs). In consistent with our results, a previous study already found that alternations of *Lachnospiraceae*, *Ruminococcaceae*, and *Enterobacteriaceae* were both observed in patients with SCZ and BD,^27^, ^43^ and *Veillonellaceae* were decreased in patients with first‐episode psychosis compared with healthy controls.^41^ This result revealed that these two diseases have overlapped intestinal microecological characteristics and which may be related to similar pathogenesis such as the change of kynurenine and short‐chain fatty acids, the important metabolites of gut microbiota.^44^, ^45^ On the other hand, SCH‐specific OTUs mainly belongs to *Lachnospiraceae* (9 OTUs), *Bacteroidaceae* (5 OTUs), *Enterobacteriaceae* (4 OTUs), *Eubacterium_coprostanoligenes_group* (3 OTUs), and *Prevotellaceae* (3 OTUs), whereas BD‐specific OTUs were mainly belongs to *Lachnospiraceae* (13 OTUs), *Oscillospiraceae* (4 OTUs), *norank_o_Clostridia_UCG‐014* (3 OTUs) and *Prevotellaceae* (3 OTUs). This difference might reveal the different intestinal microecological mechanisms between SCZ and BD. Due to different taxa of the same family might be associated with different intra‐ and extraintestinal diseases,^46^ the functions of different OTUs in the same family still need to be further studied.

BD patients showed slow thinking, lack of motivation, and reduced activity in the depressive phase, whereas schizophrenia patients with negative symptoms also have similar performance. Moreover, bipolar manic or hypomanic may have exaggerated delusions, irritability, and other manifestations, which may also exist in schizophrenia patients.^47^ However, little is known about the difference between subtypes of BD and SCH from the perspective of gut microbiota. The present study investigated the differences in the microbial signatures and function abundance between subtypes of BD and SCH. We found that 9 genera and Wnt signaling pathway were enriched in the BD‐D group, whereas 5 genera and metabolism‐related pathways were enriched in the SCH‐N group. Meanwhile, 6 genera and metabolism‐related pathways were enriched in the BD‐M group, whereas 4 genera were enriched in the SCH‐P group. These results suggested that the microbiome involved in metabolic regulation function has positive significance in distinguishing similar symptoms of SCH and BD. Furthermore, we identified 16 genera that could effectively distinguish BD‐D from SCH‐N (AUC = 0.969), and 31 genera could effectively distinguish BD‐M from SCH‐P (AUC = 0.938). We speculate that this method has the potential as a supplement for clinical diagnosis for subgroups of BD and SCH, which needs to be verified by larger samples in the future.

Nevertheless, accumulated evidence has reported the interactions between antidepressants or antipsychotics and gut microbiota.^48^, ^49^ Although we excluded patients who took any type of psychotropic drugs continuously for more than 3 days in the 2 weeks before the start of the study, the influence of antidepressant and antipsychotic drugs on the microbiome cannot be excluded. Furthermore, the present study only identified microbial panels in emerging adults with BD and SCH. Of note, the age and sex differences of the microbiome should not be ignored.^50^, ^51^ Therefore, these potential makers could not reflect the characteristics of gut microbiota in patients under other age conditions and sex composition. In addition, other signatures that can discriminate patients with BD and SCH from HCs and each other, such as inflammation, oxidative stress, and lipid composition have been recently reported,^52^, ^53^ and the interaction between the gut microbiome and these signatures needs to be further explored.

Several limitations of this study should be mentioned. First, the number of recruited participants was relatively small, especially in the subtypes of BD and SCZ. Second, our findings failed to show the diagnostic accuracy of microbial panels on disease development, a limitation that is inherent to the cross‐sectional nature. Moreover, it is necessary to exclude the influence of drugs on gut microbiome by including patients with primary untreated BD or SCH, and a discovery and validation cohort study with larger sample size and patients with a different disease course should be conducted in the future.

## 5 CONCLUSIONS

In summary, herein we have characterized and identified different gut microbiota compositions in emerging adults with BD, SCH, and their subtypes. Notably, gut microbial markers might be helpful for classifying BD and SCH as well as their subtypes with similar symptoms. These findings provide further evidence that the microbiota–gut–brain axis is involved in the pathogenesis of BD and SCH and lay the potential foundation for further development of microbe‐based clinical diagnosis for BD and SCH.

## CONFLICT OF INTEREST

The authors have no conflicts of interest to declare.

## Supporting information


Figure S1

Figure S2

Figure S3

Table S1

Table S2

Table S3

Table S4

Table S5

Table S6

Table S7

## Data Availability

The data that support the findings of this study are available from the corresponding author upon reasonable request.
