# Supplementary figures and images for: Gut microbial signatures and differences in bipolar disorder and schizophrenia of emerging adulthood
Source: CNS Neurosci Ther. 2022 Dec 5;29(Suppl 1):5–17. doi: 10.1111/cns.14044 (PMC10314106; doi:10.1111/cns.14044)

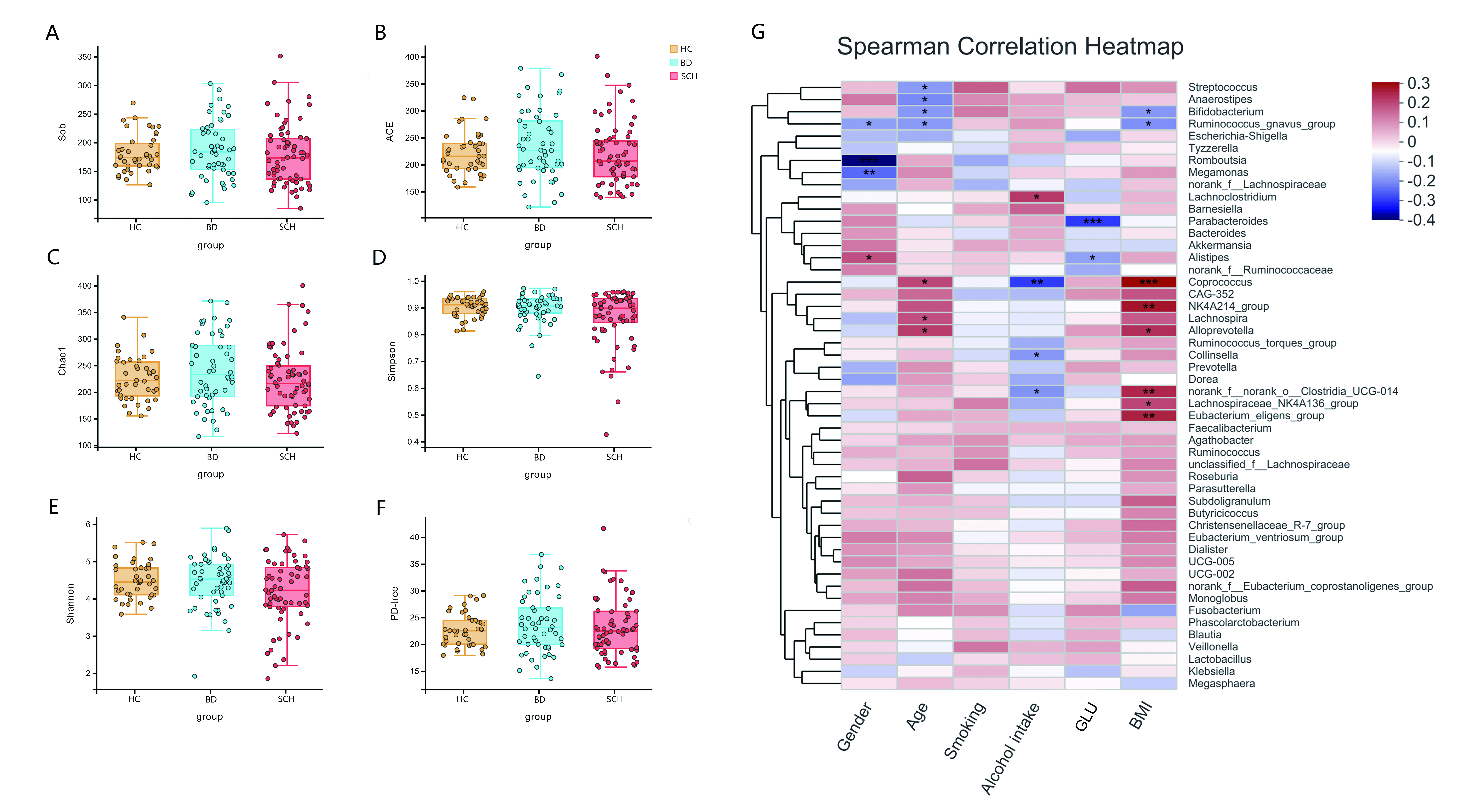

Supplement: Supplementary file 1 — Figure S1 [file CNS-29-5-s008.jpg]

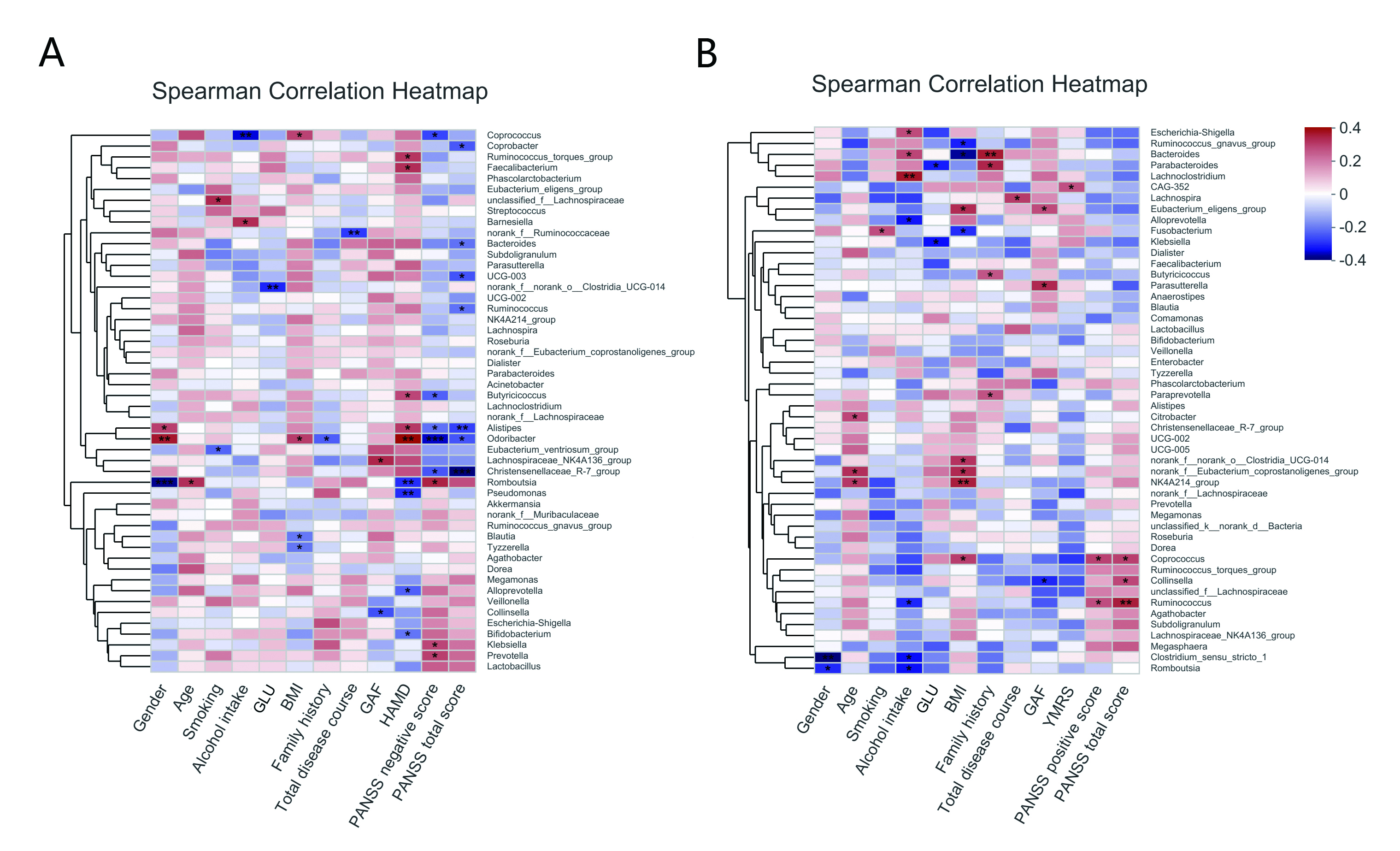

Supplement: Supplementary file 2 — Figure S2 [file CNS-29-5-s009.jpg]

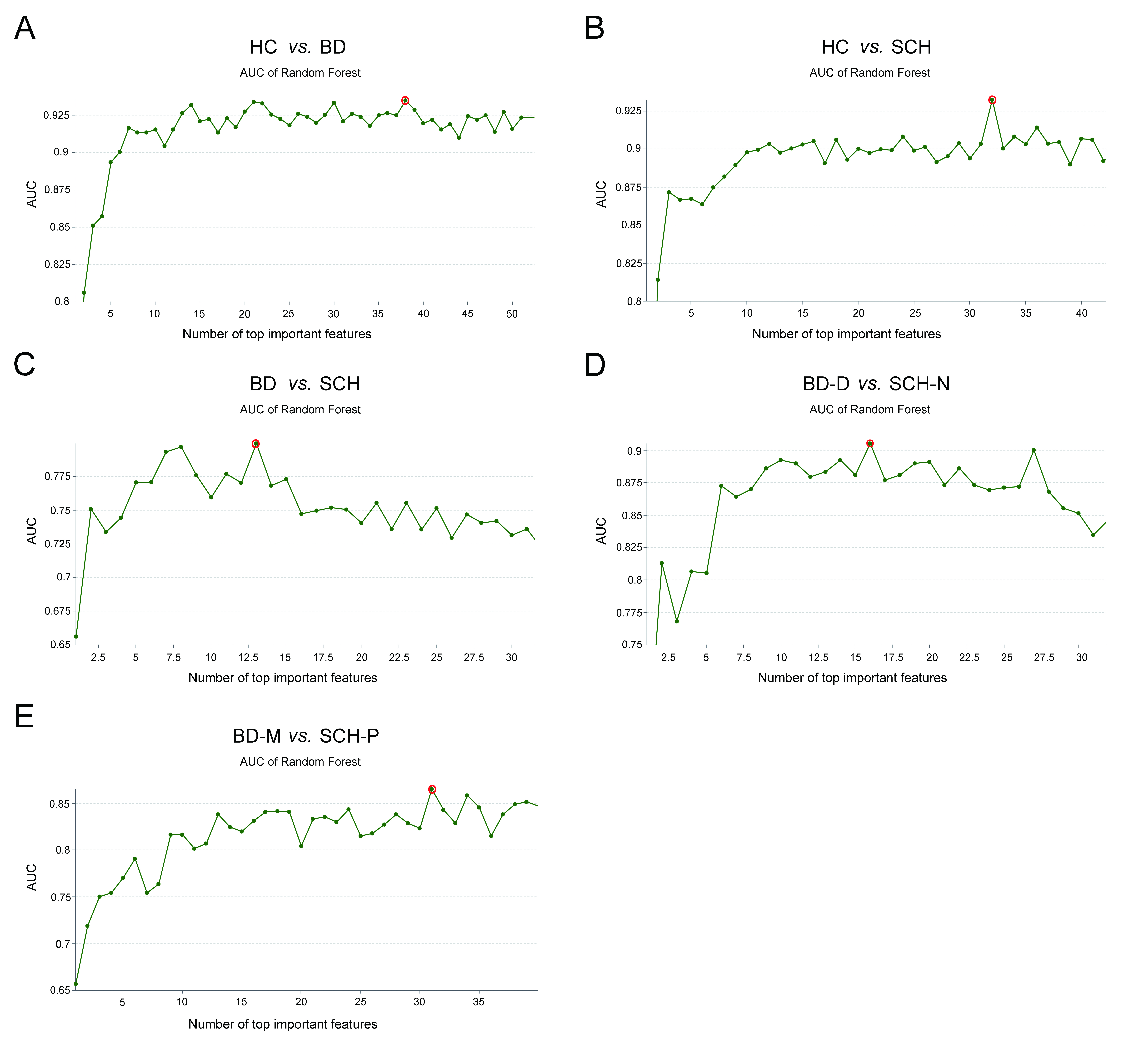

Supplement: Supplementary file 3 — Figure S3 [file CNS-29-5-s003.jpg]
