# Supplementary material for: Gut microbial signatures and differences in bipolar disorder and schizophrenia of emerging adulthood
Source: CNS Neurosci Ther. 2022 Dec 5;29(Suppl 1):5–17. doi: 10.1111/cns.14044 (PMC10314106; doi:10.1111/cns.14044)
Supplement: Supplementary file 5 — Table S2 [file CNS-29-5-s002.docx]

Supplementary Table 2. Differences in gut microbiota among BD, SCH and HC groups at the phylum, family and genus levels

| Different levels of gut microbiota | Relative abundance | | | The proportion of abundance | | | Enrichment | | *P*-value |
| --- | --- | --- | --- | --- | --- | --- | --- | --- | --- |
|  | HC | BD | SCH | HC ratio (%) | BD ratio (%) | SCH ratio (%) |  |  |  |
| **At the phylum level** | | | | | | | | | |
| Firmicutes | 62.760 | 45.891 | 48.001 | 40.063 | 29.295 | 30.642 | | HC | 0.000 |
| Actinobacteria | 3.401 | 1.460 | 2.339 | 47.236 | 20.275 | 32.489 | | HC | 0.038 |
| Proteobacteria | 2.710 | 11.896 | 10.836 | 10.652 | 46.757 | 42.591 | | BD | 0.000 |
| Epsilonbacteraeota | 0.000 | 0.011 | 0.004 | 2.899 | 72.224 | 24.877 | | BD | 0.009 |
| Bacteroidetes | 30.769 | 39.856 | 37.446 | 28.471 | 36.879 | 34.650 | | BD | 0.022 |
| Synergistetes | 0.008 | 0.056 | 0.003 | 11.554 | 83.490 | 4.956 | | BD | 0.031 |
| Euryarchaeota | 0.000 | 0.133 | 0.261 | 0.022 | 33.772 | 66.206 | | SCH | 0.014 |
| **At the family level** | | | | | | | | | |
| Ruminococcaceae | 29.067 | 22.283 | 19.230 | 41.183 | 31.572 | 27.245 | | HC | 0.000 |
| Lachnospiraceae | 20.200 | 15.324 | 15.084 | 39.914 | 30.281 | 29.805 | | HC | 0.000 |
| Veillonellaceae | 10.200 | 4.175 | 9.284 | 43.114 | 17.647 | 39.240 | | HC | 0.005 |
| Rhizobiaceae | 0.011 | 0.007 | 0.009 | 40.682 | 26.628 | 32.690 | | HC | 0.007 |
| Caulobacteraceae | 0.062 | 0.040 | 0.041 | 43.273 | 28.027 | 28.700 | | HC | 0.023 |
| Coriobacteriales_Incertae_Sedis | 0.014 | 0.011 | 0.007 | 44.256 | 34.472 | 21.272 | | HC | 0.045 |
| Burkholderiaceae | 0.568 | 1.910 | 0.776 | 17.465 | 58.688 | 23.847 | | BD | 0.000 |
| Enterobacteriaceae | 1.442 | 8.802 | 8.800 | 7.572 | 46.219 | 46.209 | | BD | 0.000 |
| Marinifilaceae | 0.194 | 0.478 | 0.392 | 18.249 | 44.902 | 36.850 | | BD | 0.007 |
| Neisseriaceae | 0.001 | 0.007 | 0.001 | 10.807 | 80.351 | 8.843 | | BD | 0.009 |
| Arcobacteraceae | 0.000 | 0.008 | 0.000 | 0.000 | 100.000 | 0.000 | | BD | 0.015 |
| Synergistaceae | 0.008 | 0.056 | 0.003 | 11.554 | 83.490 | 4.956 | | BD | 0.031 |
| Bacteroidaceae | 19.519 | 28.212 | 21.756 | 28.090 | 40.601 | 31.309 | | BD | 0.035 |
| Rhodobacteraceae | 0.001 | 0.004 | 0.000 | 18.170 | 75.976 | 5.854 | | BD | 0.048 |
| Methanobacteriaceae | 0.000 | 0.133 | 0.261 | 0.022 | 33.772 | 66.206 | | SCH | 0.014 |
| Enterococcaceae | 0.002 | 0.009 | 0.019 | 7.934 | 28.607 | 63.459 | | SCH | 0.018 |
| Eggerthellaceae | 0.115 | 0.067 | 0.164 | 33.171 | 19.303 | 47.526 | | SCH | 0.026 |
| **At the genus level** | | | | | | | | | |
| Faecalibacterium | 13.402 | 11.054 | 8.078 | 41.193 | 33.978 | 24.830 | | HC | 0.001 |
| Agathobacter | 6.521 | 2.765 | 3.835 | 49.699 | 21.071 | 29.229 | | HC | 0.000 |
| Subdoligranulum | 3.148 | 1.904 | 2.348 | 42.544 | 25.729 | 31.726 | | HC | 0.001 |
| Ruminococcus_2 | 3.346 | 1.491 | 1.556 | 52.340 | 23.319 | 24.341 | | HC | 0.000 |
| Blautia | 1.422 | 0.855 | 0.970 | 43.782 | 26.340 | 29.878 | | HC | 0.041 |
| Ruminococcus_torques_group | 1.350 | 0.900 | 0.819 | 43.981 | 29.330 | 26.689 | | HC | 0.035 |
| Lachnospiraceae_NK4A136_group | 1.359 | 0.909 | 0.637 | 46.797 | 31.275 | 21.928 | | HC | 0.001 |
| Ruminococcus_1 | 1.352 | 0.579 | 0.423 | 57.439 | 24.582 | 17.979 | | HC | 0.001 |
| Collinsella | 1.181 | 0.288 | 0.495 | 60.123 | 14.663 | 25.214 | | HC | 0.000 |
| Ruminococcaceae_UCG-014 | 0.921 | 0.290 | 0.625 | 50.168 | 15.814 | 34.018 | | HC | 0.020 |
| Dorea | 0.939 | 0.340 | 0.472 | 53.630 | 19.429 | 26.940 | | HC | 0.000 |
| Butyricicoccus | 0.653 | 0.447 | 0.326 | 45.796 | 31.356 | 22.848 | | HC | 0.000 |
| Ruminococcaceae_UCG-013 | 0.726 | 0.254 | 0.215 | 60.803 | 21.239 | 17.958 | | HC | 0.000 |
| Coprococcus_3 | 0.477 | 0.139 | 0.181 | 59.826 | 17.501 | 22.672 | | HC | 0.000 |
| Tyzzerella_3 | 0.414 | 0.230 | 0.095 | 56.036 | 31.119 | 12.845 | | HC | 0.005 |
| Coprococcus_1 | 0.090 | 0.043 | 0.045 | 50.815 | 23.949 | 25.235 | | HC | 0.009 |
| Lachnospiraceae_ND3007_group | 0.033 | 0.029 | 0.025 | 38.284 | 32.787 | 28.929 | | HC | 0.015 |
| Erysipelotrichaceae_UCG-003 | 0.042 | 0.014 | 0.022 | 54.205 | 17.698 | 28.096 | | HC | 0.000 |
| Lachnospiraceae_FCS020_group | 0.026 | 0.012 | 0.014 | 49.385 | 23.376 | 27.239 | | HC | 0.002 |
| Slackia | 0.044 | 0.002 | 0.007 | 83.895 | 3.219 | 12.885 | | HC | 0.002 |
| Adlercreutzia | 0.018 | 0.007 | 0.009 | 52.440 | 19.873 | 27.687 | | HC | 0.008 |
| Allorhizobium-Neorhizobium-Pararhizobium-Rhizobium | 0.009 | 0.007 | 0.008 | 37.140 | 27.855 | 35.006 | | HC | 0.004 |
| Alkanindiges | 0.007 | 0.000 | 0.000 | 93.076 | 4.101 | 2.823 | | HC | 0.048 |
| Bacteroides | 19.519 | 28.212 | 21.756 | 28.090 | 40.601 | 31.309 | | BD | 0.035 |
| Escherichia-Shigella | 1.020 | 7.429 | 7.016 | 6.596 | 48.037 | 45.367 | | BD | 0.000 |
| Parasutterella | 0.441 | 0.957 | 0.534 | 22.834 | 49.535 | 27.632 | | BD | 0.001 |
| Eubacterium_eligens_group | 0.571 | 0.809 | 0.407 | 31.930 | 45.291 | 22.778 | | BD | 0.018 |
| Odoribacter | 0.110 | 0.327 | 0.268 | 15.563 | 46.449 | 37.989 | | BD | 0.003 |
| Fusicatenibacter | 0.215 | 0.305 | 0.208 | 29.512 | 41.922 | 28.566 | | BD | 0.004 |
| Bilophila | 0.096 | 0.233 | 0.150 | 19.956 | 48.715 | 31.329 | | BD | 0.001 |
| Oscillibacter | 0.077 | 0.283 | 0.115 | 16.173 | 59.683 | 24.144 | | BD | 0.008 |
| Flavonifractor | 0.080 | 0.202 | 0.132 | 19.357 | 48.780 | 31.863 | | BD | 0.004 |
| Turicibacter | 0.020 | 0.164 | 0.028 | 9.510 | 77.257 | 13.233 | | BD | 0.009 |
| Prevotella | 0.014 | 0.069 | 0.051 | 10.645 | 51.566 | 37.789 | | BD | 0.010 |
| Cloacibacillus | 0.001 | 0.046 | 0.001 | 2.289 | 94.729 | 2.982 | | BD | 0.019 |
| Anaerotruncus | 0.004 | 0.017 | 0.006 | 12.879 | 63.597 | 23.523 | | BD | 0.007 |
| Blastomonas | 0.000 | 0.011 | 0.006 | 1.979 | 64.699 | 33.322 | | BD | 0.006 |
| Arcobacter | 0.000 | 0.008 | 0.000 | 0.000 | 100.000 | 0.000 | | BD | 0.015 |
| Paracoccus | 0.000 | 0.004 | 0.000 | 0.000 | 92.847 | 7.153 | | BD | 0.005 |
| Uruburuella | 0.000 | 0.004 | 0.000 | 0.000 | 100.000 | 0.000 | | BD | 0.044 |
| Romboutsia | 0.372 | 0.193 | 0.530 | 33.991 | 17.635 | 48.374 | | SCH | 0.011 |
| Lachnospiraceae_UCG-004 | 0.074 | 0.244 | 0.313 | 11.790 | 38.606 | 49.604 | | SCH | 0.006 |
| Methanobrevibacter | 0.000 | 0.133 | 0.261 | 0.022 | 33.772 | 66.206 | | SCH | 0.014 |
| Prevotellaceae_NK3B31_group | 0.000 | 0.038 | 0.250 | 0.000 | 13.209 | 86.791 | | SCH | 0.000 |
| Prevotellaceae_UCG-001 | 0.000 | 0.024 | 0.136 | 0.060 | 14.761 | 85.179 | | SCH | 0.001 |
| Cronobacter | 0.002 | 0.014 | 0.081 | 1.590 | 14.572 | 83.837 | | SCH | 0.014 |
| Enterococcus | 0.002 | 0.009 | 0.019 | 7.371 | 28.591 | 64.038 | | SCH | 0.019 |
| Sphingopyxis | 0.004 | 0.003 | 0.009 | 22.788 | 20.135 | 57.078 | | SCH | 0.036 |
