# Supplementary material for: Gut microbial signatures and differences in bipolar disorder and schizophrenia of emerging adulthood
Source: CNS Neurosci Ther. 2022 Dec 5;29(Suppl 1):5–17. doi: 10.1111/cns.14044 (PMC10314106; doi:10.1111/cns.14044)
Supplement: Supplementary file 7 — Table S4 [file CNS-29-5-s007.docx]

Supplementary Table 4. Comparison of clinical characteristics data and alpha diversity estimation between the BD-D subgroup and the SCH-N subgroup

| Parameter | BD - D (n = 30) | SCH - N (n = 26) | *t / Z / χ^2^* *- value* | *P*-value |
| --- | --- | --- | --- | --- |
| Family history (Yes / No) ^c^ | 8/22 | 8/18 | χ^2^ = 0.115 | 0.735 |
| Total disease course (months) ^b^ | 42.00 (19.50, 60.00) | 24.00 (10.00, 45.00) | Z = -1.407 | 0.160 |
| HAMD (means ± SD) ^a^ | 25.97 ± 4.41 (18.00 - 36.00) | 15.88 ± 2.75 (9.00 - 20.00) | t = 10.086 | <0.001 |
| PANSS negative score (means ± SD) ^a^ | 16.17 ± 2.83 (10.00 - 21.00) | 24.77 ± 3.08 (22.00 - 33.00) | t = -10.897 | <0.001 |
| PANSS total score [M (P25, P75)] ^b^ | 59.00 (55.00, 77.50) | 80.00 (74.50, 85.25) | Z = -4.375 | <0.001 |
| GAF [M (P25, P75)] ^b^ | 42.50 (34.25, 47.25) | 36.50 (33.50, 41.25) | Z = -2.262 | 0.024 |
| Sobs ^a^ | 198.67 ± 46.58 (109.00-303.00) | 182.27 ± 45.30 (116.00 - 268.00) | t = 1.331 | 0.189 |
| Ace ^a^ | 247.19 ± 60.10 (142.59, 378.65) | 227.88 ± 51.34 (144.79, 347.10) | t = 1.283 | 0.205 |
| Chao1 ^a^ | 252.03 ± 62.36 (145.09, 371.17) | 230.76 ± 54.20 (143.00, 362.67) | t = 1.352 | 0.182 |
| Shannon ^a^ | 4.59 ± 0.63 (3.38, 5.89) | 4.19 ± 0.85 (2.20, 5.36) | t = 1.977 | 0.053 |
| Simpson ^b^ | 0.91 (0.89, 0.94) | 0.87 (0.85, 0.94) | Z=-1.462 | 0.144 |
| PD-whole tree ^a^ | 24.86 ± 5.06 (15.08, 36.74) | 23.41 ± 4.85 (16.07, 31.84) | t = 1.084 | 0.283 |

Abbreviations: ^a^ Student's t-test; ^b^ Mann-Whitney U; ^c^ Chi-square analysis; Sob, Observer-species
