# Supplementary material for: Gut microbial signatures and differences in bipolar disorder and schizophrenia of emerging adulthood
Source: CNS Neurosci Ther. 2022 Dec 5;29(Suppl 1):5–17. doi: 10.1111/cns.14044 (PMC10314106; doi:10.1111/cns.14044)
Supplement: Supplementary file 8 — Table S5 [file CNS-29-5-s005.docx]

Supplementary Table 5. Differences in bacterial function between the HC and each subgroup (BD-D, BD-M, SCH-N and SCH-P), BD-D and SCH-N, BD-M and SCH-P subgroups

| Comparison | Pathway abundance in | Level_1 | Level_2 | Level_3 | *P*-value |
| --- | --- | --- | --- | --- | --- |
| HC *vs.* BD-D | BD-D | Cellular Processes | Cell growth and death | Meiosis - yeast | 0.029681 |
|  | BD-D | Environmental Information Processing | Membrane transport | ABC transporters | 0.014553 |
|  | BD-D | Environmental Information Processing | Signaling molecules and interaction | ECM-receptor interaction | 0.03775 |
|  | BD-D | Genetic Information Processing | Folding, sorting and degradation | Proteasome | 0.003291 |
|  | BD-D | Genetic Information Processing | Folding, sorting and degradation | Protein processing in endoplasmic reticulum | 0.041284 |
|  | BD-D | Genetic Information Processing | Replication and repair | Non-homologous end-joining | 0.001918 |
|  | BD-D | Genetic Information Processing | Replication and repair | Nucleotide excision repair | 0.005081 |
|  | BD-D | Genetic Information Processing | Replication and repair | Base excision repair | 0.007692 |
|  | BD-D | Genetic Information Processing | Transcription | RNA polymerase | 0.042505 |
|  | BD-D | Human Diseases | Cardiovascular diseases | Hypertrophic cardiomyopathy (HCM) | 3.35E-05 |
| HC *vs.* BD-D | BD-D | Human Diseases | Infectious diseases | Epithelial cell signaling in Helicobacter pylori infection | 0.015055 |
|  | BD-D | Human Diseases | Infectious diseases | Bacterial invasion of epithelial cells | 0.024139 |
|  | BD-D | Metabolism | Amino acid metabolism | Lysine degradation | 0.009916 |
|  | BD-D | Metabolism | Amino acid metabolism | Phenylalanine metabolism | 0.010647 |
|  | BD-D | Metabolism | Amino acid metabolism | Tryptophan metabolism | 0.012252 |
|  | BD-D | Metabolism | Amino acid metabolism | Tyrosine metabolism | 0.013591 |
|  | BD-D | Metabolism | Amino acid metabolism | Arginine and proline metabolism | 0.016106 |
|  | BD-D | Metabolism | Amino acid metabolism | Cysteine and methionine metabolism | 0.019015 |
|  | BD-D | Metabolism | Amino acid metabolism | Histidine metabolism | 0.022367 |
|  | BD-D | Metabolism | Amino acid metabolism | Valine, leucine and isoleucine degradation | 0.040092 |
|  | BD-D | Metabolism | Biosynthesis of other secondary metabolites | Betalain biosynthesis | 0.000687 |
|  | BD-D | Metabolism | Biosynthesis of other secondary metabolites | Penicillin and cephalosporin biosynthesis | 0.00316 |
|  | BD-D | Metabolism | Biosynthesis of other secondary metabolites | Tropane, piperidine and pyridine alkaloid biosynthesis | 0.00798 |
|  | BD-D | Metabolism | Biosynthesis of other secondary metabolites | Streptomycin biosynthesis | 0.009916 |
|  | HC | Metabolism | Biosynthesis of other secondary metabolites | Clavulanic acid biosynthesis | 0.032167 |
|  | BD-D | Metabolism | Carbohydrate metabolism | Inositol phosphate metabolism | 0.001687 |
|  | BD-D | Metabolism | Carbohydrate metabolism | Galactose metabolism | 0.004888 |
|  |  |  |  |  |  |
| HC *vs.* BD-D | BD-D | Metabolism | Carbohydrate metabolism | Ascorbate and aldarate metabolism | 0.005921 |
|  | BD-D | Metabolism | Carbohydrate metabolism | Starch and sucrose metabolism | 0.00663 |
|  | BD-D | Metabolism | Carbohydrate metabolism | Propanoate metabolism | 0.014553 |
|  | BD-D | Metabolism | Carbohydrate metabolism | Citrate cycle (TCA cycle) | 0.021659 |
|  | BD-D | Metabolism | Carbohydrate metabolism | Glycolysis / Gluconeogenesis | 0.021659 |
|  | BD-D | Metabolism | Carbohydrate metabolism | Butanoate metabolism | 0.022367 |
|  | BD-D | Metabolism | Carbohydrate metabolism | Pentose phosphate pathway | 0.024611 |
|  | BD-D | Metabolism | Carbohydrate metabolism | Pentose and glucuronate interconversions | 0.0279 |
|  | BD-D | Metabolism | Carbohydrate metabolism | Fructose and mannose metabolism | 0.031556 |
|  | BD-D | Metabolism | Carbohydrate metabolism | Glyoxylate and dicarboxylate metabolism | 0.034557 |
|  | BD-D | Metabolism | Carbohydrate metabolism | C5-Branched dibasic acid metabolism | 0.036689 |
|  | BD-D | Metabolism | Energy metabolism | Methane metabolism | 0.009916 |
|  | BD-D | Metabolism | Energy metabolism | Carbon fixation pathways in prokaryotes | 0.040092 |
|  | BD-D | Metabolism | Energy metabolism | Oxidative phosphorylation | 0.04907 |
|  | BD-D | Metabolism | Glycan biosynthesis and metabolism | N-Glycan biosynthesis | 0.012252 |
|  | BD-D | Metabolism | Lipid metabolism | Steroid biosynthesis | 0.002002 |
|  | BD-D | Metabolism | Lipid metabolism | Primary bile acid biosynthesis | 0.0057 |
|  | BD-D | Metabolism | Lipid metabolism | Fatty acid degradation | 0.012685 |
|  | BD-D | Metabolism | Lipid metabolism | Sphingolipid metabolism | 0.0203 |
|  | BD-D | Metabolism | Lipid metabolism | Glycerolipid metabolism | 0.02097 |
| HC *vs.* BD-D | BD-D | Metabolism | Lipid metabolism | Synthesis and degradation of ketone bodies | 0.025401 |
|  | BD-D | Metabolism | Lipid metabolism | Linoleic acid metabolism | 0.032531 |
|  | BD-D | Metabolism | Metabolism of cofactors and vitamins | Porphyrin and chlorophyll metabolism | 0.015055 |
|  | BD-D | Metabolism | Metabolism of cofactors and vitamins | Riboflavin metabolism | 0.036689 |
|  | BD-D | Metabolism | Metabolism of cofactors and vitamins | Pantothenate and CoA biosynthesis | 0.04907 |
|  | BD-D | Metabolism | Metabolism of other amino acids | Cyanoamino acid metabolism | 0.001761 |
|  | BD-D | Metabolism | Metabolism of other amino acids | beta-Alanine metabolism | 0.002683 |
|  | BD-D | Metabolism | Metabolism of other amino acids | Taurine and hypotaurine metabolism | 0.00798 |
|  | BD-D | Metabolism | Metabolism of other amino acids | Selenocompound metabolism | 0.01722 |
|  | BD-D | Metabolism | Metabolism of terpenoids and polyketides | Sesquiterpenoid and triterpenoid biosynthesis | 0.001188 |
|  | BD-D | Metabolism | Metabolism of terpenoids and polyketides | Limonene and pinene degradation | 0.005921 |
|  | BD-D | Metabolism | Metabolism of terpenoids and polyketides | Terpenoid backbone biosynthesis | 0.009229 |
|  | BD-D | Metabolism | Metabolism of terpenoids and polyketides | Biosynthesis of siderophore group nonribosomal peptides | 0.015055 |
| HC *vs.* BD-D | BD-D | Metabolism | Metabolism of terpenoids and polyketides | Biosynthesis of type II polyketide backbone | 0.015573 |
|  | BD-D | Metabolism | Metabolism of terpenoids and polyketides | Geraniol degradation | 0.019015 |
|  | BD-D | Metabolism | Metabolism of terpenoids and polyketides | Polyketide sugar unit biosynthesis | 0.03893 |
|  | BD-D | Metabolism | Xenobiotics biodegradation and metabolism | Fluorobenzoate degradation | 0.001086 |
|  | BD-D | Metabolism | Xenobiotics biodegradation and metabolism | Aminobenzoate degradation | 0.001136 |
|  | BD-D | Metabolism | Xenobiotics biodegradation and metabolism | Benzoate degradation | 0.001547 |
|  | BD-D | Metabolism | Xenobiotics biodegradation and metabolism | Chlorocyclohexane and chlorobenzene degradation | 0.001918 |
|  | BD-D | Metabolism | Xenobiotics biodegradation and metabolism | Dioxin degradation | 0.002272 |
|  | BD-D | Metabolism | Xenobiotics biodegradation and metabolism | Toluene degradation | 0.004002 |
|  | BD-D | Metabolism | Xenobiotics biodegradation and metabolism | Chloroalkane and chloroalkene degradation | 0.006386 |
|  | BD-D | Metabolism | Xenobiotics biodegradation and metabolism | Caprolactam degradation | 0.007414 |
|  | BD-D | Metabolism | Xenobiotics biodegradation and metabolism | Styrene degradation | 0.009567 |
|  | BD-D | Metabolism | Xenobiotics biodegradation and metabolism | Xylene degradation | 0.019377 |
| HC *vs.* BD-D | BD-D | Metabolism | Xenobiotics biodegradation and metabolism | Metabolism of xenobiotics by cytochrome P450 | 0.034577 |
|  | BD-D | Metabolism | Xenobiotics biodegradation and metabolism | Bisphenol degradation | 0.034877 |
|  | BD-D | Organismal Systems | Digestive system | Protein digestion and absorption | 0.033531 |
|  | BD-D | Organismal Systems | Immune system | NOD-like receptor signaling pathway | 0.003426 |
|  | | | | | |
| HC *vs.* BD-M | BD-M | Metabolism | Amino acid metabolism | Lysine degradation | 0.001851 |
|  | BD-M | Metabolism | Amino acid metabolism | Tryptophan metabolism | 0.001961 |
|  | BD-M | Metabolism | Amino acid metabolism | Phenylalanine metabolism | 0.0061 |
|  | BD-M | Metabolism | Amino acid metabolism | Tyrosine metabolism | 0.008227 |
|  | BD-M | Metabolism | Amino acid metabolism | Valine, leucine and isoleucine biosynthesis | 0.008638 |
|  | BD-M | Metabolism | Amino acid metabolism | Histidine metabolism | 0.012634 |
|  | BD-M | Metabolism | Amino acid metabolism | Valine, leucine and isoleucine degradation | 0.013854 |
|  | BD-M | Metabolism | Amino acid metabolism | Cysteine and methionine metabolism | 0.025605 |
|  | BD-M | Metabolism | Amino acid metabolism | Arginine and proline metabolism | 0.025605 |
|  | BD-M | Metabolism | Biosynthesis of other secondary metabolites | Streptomycin biosynthesis | 7.04E-05 |
|  | BD-M | Metabolism | Biosynthesis of other secondary metabolites | Penicillin and cephalosporin biosynthesis | 0.000111 |
|  | BD-M | Metabolism | Biosynthesis of other secondary metabolites | Betalain biosynthesis | 0.000343 |
| HC *vs.* BD-M | BD-M | Metabolism | Biosynthesis of other secondary metabolites | Tropane, piperidine and pyridine alkaloid biosynthesis | 0.010471 |
|  | BD-M | Metabolism | Carbohydrate metabolism | Galactose metabolism | 0.000279 |
|  | BD-M | Metabolism | Carbohydrate metabolism | Inositol phosphate metabolism | 0.000581 |
|  | BD-M | Metabolism | Carbohydrate metabolism | Starch and sucrose metabolism | 0.001381 |
|  | BD-M | Metabolism | Carbohydrate metabolism | Ascorbate and aldarate metabolism | 0.001648 |
|  | BD-M | Metabolism | Carbohydrate metabolism | Citrate cycle (TCA cycle) | 0.007093 |
|  | BD-M | Metabolism | Carbohydrate metabolism | Propanoate metabolism | 0.012634 |
|  | BD-M | Metabolism | Carbohydrate metabolism | Butanoate metabolism | 0.013232 |
|  | BD-M | Metabolism | Carbohydrate metabolism | Fructose and mannose metabolism | 0.013854 |
|  | BD-M | Metabolism | Carbohydrate metabolism | C5-Branched dibasic acid metabolism | 0.015875 |
|  | BD-M | Metabolism | Carbohydrate metabolism | Glycolysis / Gluconeogenesis | 0.018961 |
|  | BD-M | Metabolism | Carbohydrate metabolism | Pentose and glucuronate interconversions | 0.021601 |
|  | BD-M | Metabolism | Carbohydrate metabolism | Pentose phosphate pathway | 0.027832 |
|  | BD-M | Metabolism | Carbohydrate metabolism | Amino sugar and nucleotide sugar metabolism | 0.027832 |
|  | BD-M | Metabolism | Carbohydrate metabolism | Glyoxylate and dicarboxylate metabolism | 0.031479 |
|  | BD-M | Metabolism | Energy metabolism | Carbon fixation pathways in prokaryotes | 0.01206 |
|  | BD-M | Metabolism | Energy metabolism | Methane metabolism | 0.044911 |
|  | BD-M | Metabolism | Glycan biosynthesis and metabolism | N-Glycan biosynthesis | 0.001648 |
| HC *vs.* BD-M | BD-M | Metabolism | Glycan biosynthesis and metabolism | Other types of O-glycan biosynthesis | 0.010827 |
|  | BD-M | Metabolism | Glycan biosynthesis and metabolism | Other glycan degradation | 0.01206 |
|  | BD-M | Metabolism | Lipid metabolism | Primary bile acid biosynthesis | 0.000212 |
|  | BD-M | Metabolism | Lipid metabolism | Steroid biosynthesis | 0.000212 |
|  | BD-M | Metabolism | Lipid metabolism | Fatty acid degradation | 0.001554 |
|  | BD-M | Metabolism | Lipid metabolism | Glycerolipid metabolism | 0.003426 |
|  | BD-M | Metabolism | Lipid metabolism | Synthesis and degradation of ketone bodies | 0.005507 |
|  | BD-M | Metabolism | Lipid metabolism | Sphingolipid metabolism | 0.006416 |
|  | BD-M | Metabolism | Lipid metabolism | Linoleic acid metabolism | 0.015875 |
|  | BD-M | Metabolism | Metabolism of cofactors and vitamins | Biotin metabolism | 0.006416 |
|  | BD-M | Metabolism | Metabolism of cofactors and vitamins | Pantothenate and CoA biosynthesis | 0.007093 |
|  | BD-M | Metabolism | Metabolism of cofactors and vitamins | One carbon pool by folate | 0.015875 |
|  | BD-M | Metabolism | Metabolism of cofactors and vitamins | Porphyrin and chlorophyll metabolism | 0.017359 |
|  | BD-M | Metabolism | Metabolism of other amino acids | beta-Alanine metabolism | 0.000478 |
|  | BD-M | Metabolism | Metabolism of other amino acids | Cyanoamino acid metabolism | 0.002326 |
| HC *vs.* BD-M | BD-M | Metabolism | Metabolism of other amino acids | Selenocompound metabolism | 0.008638 |
|  | BD-M | Metabolism | Metabolism of other amino acids | Taurine and hypotaurine metabolism | 0.032781 |
|  | BD-M | Metabolism | Metabolism of terpenoids and polyketides | Polyketide sugar unit biosynthesis | 5.59E-05 |
|  | BD-M | Metabolism | Metabolism of terpenoids and polyketides | Sesquiterpenoid and triterpenoid biosynthesis | 0.000419 |
|  | BD-M | Metabolism | Metabolism of terpenoids and polyketides | Limonene and pinene degradation | 0.000619 |
|  | BD-M | Metabolism | Metabolism of terpenoids and polyketides | Terpenoid backbone biosynthesis | 0.000849 |
|  | BD-M | Metabolism | Metabolism of terpenoids and polyketides | Biosynthesis of type II polyketide backbone | 0.001301 |
|  | BD-M | Metabolism | Metabolism of terpenoids and polyketides | Geraniol degradation | 0.011508 |
|  | BD-M | Metabolism | Metabolism of terpenoids and polyketides | Biosynthesis of siderophore group nonribosomal peptides | 0.02353 |
|  | BD-M | Metabolism | Metabolism of terpenoids and polyketides | Carotenoid biosynthesis | 0.029006 |
|  | BD-M | Metabolism | Metabolism of terpenoids and polyketides | Biosynthesis of vancomycin group antibiotics | 0.03552 |
|  | BD-M | Metabolism | Xenobiotics biodegradation and metabolism | Benzoate degradation | 0.00032 |
|  | BD-M | Metabolism | Xenobiotics biodegradation and metabolism | Chlorocyclohexane and chlorobenzene degradation | 0.00032 |
| HC *vs.* BD-M | BD-M | Metabolism | Xenobiotics biodegradation and metabolism | Aminobenzoate degradation | 0.000544 |
|  | BD-M | Metabolism | Xenobiotics biodegradation and metabolism | Dioxin degradation | 0.002602 |
|  | BD-M | Metabolism | Xenobiotics biodegradation and metabolism | Caprolactam degradation | 0.003244 |
|  | BD-M | Metabolism | Xenobiotics biodegradation and metabolism | Chloroalkane and chloroalkene degradation | 0.003426 |
|  | BD-M | Metabolism | Xenobiotics biodegradation and metabolism | Fluorobenzoate degradation | 0.003616 |
|  | BD-M | Metabolism | Xenobiotics biodegradation and metabolism | Toluene degradation | 0.004212 |
|  | BD-M | Metabolism | Xenobiotics biodegradation and metabolism | Styrene degradation | 0.005797 |
|  | BD-M | Metabolism | Xenobiotics biodegradation and metabolism | Bisphenol degradation | 0.013224 |
|  | BD-M | Metabolism | Xenobiotics biodegradation and metabolism | Nitrotoluene degradation | 0.014501 |
|  | BD-M | Organismal Systems | Digestive system | Protein digestion and absorption | 0.000544 |
|  | BD-M | Organismal Systems | Endocrine system | Insulin signaling pathway | 0.032781 |
|  | BD-M | Organismal Systems | Immune system | NOD-like receptor signaling pathway | 0.004473 |
|  | BD-M | Cellular Processes | Cell growth and death | Meiosis - yeast | 0.000366 |
|  | BD-M | Environmental Information Processing | Membrane transport | ABC transporters | 0.031479 |
|  |  |  |  |  |  |
| HC *vs.* BD-M | BD-M | Genetic Information Processing | Folding, sorting and degradation | Proteasome | 0.00051 |
|  | BD-M | Genetic Information Processing | Folding, sorting and degradation | Protein processing in endoplasmic reticulum | 0.003426 |
|  | BD-M | Genetic Information Processing | Replication and repair | Nucleotide excision repair | 0.00032 |
|  | BD-M | Genetic Information Processing | Replication and repair | Non-homologous end-joining | 0.000798 |
|  | BD-M | Genetic Information Processing | Replication and repair | Base excision repair | 0.002461 |
|  | BD-M | Genetic Information Processing | Transcription | RNA polymerase | 0.0061 |
|  | BD-M | Human Diseases | Cardiovascular diseases | Hypertrophic cardiomyopathy (HCM) | 0.010804 |
|  | BD-M | Human Diseases | Infectious diseases | Bacterial invasion of epithelial cells | 7.60E-05 |
|  | BD-M | Human Diseases | Infectious diseases | Epithelial cell signaling in Helicobacter pylori infection | 0.018961 |
|  | | | | | |
| HC *vs.* SCH-N | SCH-N | Metabolism | Amino acid metabolism | Phenylalanine, tyrosine and tryptophan biosynthesis | 0.002211 |
|  | SCH-N | Metabolism | Amino acid metabolism | Lysine biosynthesis | 0.001664 |
|  | SCH-N | Metabolism | Amino acid metabolism | Alanine, aspartate and glutamate metabolism | 0.003492 |
|  | SCH-N | Metabolism | Amino acid metabolism | Cysteine and methionine metabolism | 0.001439 |
|  | SCH-N | Metabolism | Amino acid metabolism | Glycine, serine and threonine metabolism | 0.004958 |
| HC *vs.* SCH-N | SCH-N | Metabolism | Amino acid metabolism | Valine, leucine and isoleucine biosynthesis | 6.46E-05 |
|  | SCH-N | Metabolism | Amino acid metabolism | Histidine metabolism | 0.000142 |
|  | SCH-N | Metabolism | Amino acid metabolism | Arginine and proline metabolism | 0.00016 |
|  | SCH-N | Metabolism | Amino acid metabolism | Tyrosine metabolism | 0.000336 |
|  | SCH-N | Metabolism | Amino acid metabolism | Phenylalanine metabolism | 5.34E-05 |
|  | SCH-N | Metabolism | Amino acid metabolism | Valine, leucine and isoleucine degradation | 8.80E-05 |
|  | SCH-N | Metabolism | Amino acid metabolism | Lysine degradation | 5.20E-06 |
|  | SCH-N | Metabolism | Amino acid metabolism | Tryptophan metabolism | 2.29E-05 |
|  | SCH-N | Metabolism | Biosynthesis of other secondary metabolites | Tropane, piperidine and pyridine alkaloid biosynthesis | 0.00016 |
|  | SCH-N | Metabolism | Biosynthesis of other secondary metabolites | Streptomycin biosynthesis | 4.16E-06 |
|  | SCH-N | Metabolism | Biosynthesis of other secondary metabolites | Penicillin and cephalosporin biosynthesis | 4.48E-06 |
|  | SCH-N | Metabolism | Biosynthesis of other secondary metabolites | Betalain biosynthesis | 7.97E-07 |
|  | SCH-N | Metabolism | Carbohydrate metabolism | Fructose and mannose metabolism | 0.017741 |
|  | SCH-N | Metabolism | Carbohydrate metabolism | Pyruvate metabolism | 0.006394 |
|  | SCH-N | Metabolism | Carbohydrate metabolism | Amino sugar and nucleotide sugar metabolism | 0.004354 |
|  | SCH-N | Metabolism | Carbohydrate metabolism | C5-Branched dibasic acid metabolism | 7.78E-05 |
|  | SCH-N | Metabolism | Carbohydrate metabolism | Pentose phosphate pathway | 0.003339 |
|  |  |  |  |  |  |
| HC *vs.* SCH-N | SCH-N | Metabolism | Carbohydrate metabolism | Glycolysis / Gluconeogenesis | 0.000919 |
|  | SCH-N | Metabolism | Carbohydrate metabolism | Citrate cycle (TCA cycle) | 0.000269 |
|  | SCH-N | Metabolism | Carbohydrate metabolism | Glyoxylate and dicarboxylate metabolism | 0.000106 |
|  | SCH-N | Metabolism | Carbohydrate metabolism | Pentose and glucuronate interconversions | 0.003651 |
|  | SCH-N | Metabolism | Carbohydrate metabolism | Butanoate metabolism | 0.000874 |
|  | SCH-N | Metabolism | Carbohydrate metabolism | Propanoate metabolism | 6.46E-05 |
|  | SCH-N | Metabolism | Carbohydrate metabolism | Ascorbate and aldarate metabolism | 6.87E-05 |
|  | SCH-N | Metabolism | Carbohydrate metabolism | Starch and sucrose metabolism | 0.000641 |
|  | SCH-N | Metabolism | Carbohydrate metabolism | Galactose metabolism | 0.000284 |
|  | SCH-N | Metabolism | Carbohydrate metabolism | Inositol phosphate metabolism | 3.07E-06 |
|  | SCH-N | Metabolism | Energy metabolism | Methane metabolism | 0.013652 |
|  | SCH-N | Metabolism | Energy metabolism | Carbon fixation in photosynthetic organisms | 0.008526 |
|  | SCH-N | Metabolism | Energy metabolism | Photosynthesis | 0.023188 |
|  | SCH-N | Metabolism | Energy metabolism | Sulfur metabolism | 0.016477 |
|  | SCH-N | Metabolism | Energy metabolism | Oxidative phosphorylation | 0.000227 |
|  | SCH-N | Metabolism | Energy metabolism | Carbon fixation pathways in prokaryotes | 0.000191 |
|  | SCH-N | Metabolism | Glycan biosynthesis and metabolism | Peptidoglycan biosynthesis | 0.000919 |
|  | SCH-N | Metabolism | Glycan biosynthesis and metabolism | N-Glycan biosynthesis | 3.40E-05 |
|  |  |  |  |  |  |
| HC *vs.* SCH-N | SCH-N | Metabolism | Lipid metabolism | Glycerophospholipid metabolism | 0.002916 |
|  | SCH-N | Metabolism | Lipid metabolism | Fatty acid biosynthesis | 0.000967 |
|  | SCH-N | Metabolism | Lipid metabolism | Biosynthesis of unsaturated fatty acids | 0.002542 |
|  | SCH-N | Metabolism | Lipid metabolism | Glycerolipid metabolism | 0.002211 |
|  | SCH-N | Metabolism | Lipid metabolism | Linoleic acid metabolism | 0.013141 |
|  | SCH-N | Metabolism | Lipid metabolism | Synthesis and degradation of ketone bodies | 2.61E-05 |
|  | SCH-N | Metabolism | Lipid metabolism | Fatty acid degradation | 1.15E-05 |
|  | SCH-N | Metabolism | Lipid metabolism | Primary bile acid biosynthesis | 0.000119 |
|  | SCH-N | Metabolism | Lipid metabolism | Steroid biosynthesis | 2.61E-05 |
|  | SCH-N | Metabolism | Metabolism of cofactors and vitamins | Nicotinate and nicotinamide metabolism | 0.027219 |
|  | SCH-N | Metabolism | Metabolism of cofactors and vitamins | Thiamine metabolism | 0.016477 |
|  | SCH-N | Metabolism | Metabolism of cofactors and vitamins | Biotin metabolism | 0.013652 |
|  | SCH-N | Metabolism | Metabolism of cofactors and vitamins | Lipoic acid metabolism | 0.004548 |
|  | SCH-N | Metabolism | Metabolism of cofactors and vitamins | Riboflavin metabolism | 0.004354 |
|  | SCH-N | Metabolism | Metabolism of cofactors and vitamins | Pantothenate and CoA biosynthesis | 1.87E-05 |
|  | SCH-N | Metabolism | Metabolism of cofactors and vitamins | One carbon pool by folate | 0.000318 |
| HC *vs.* SCH-N | SCH-N | Metabolism | Metabolism of cofactors and vitamins | Porphyrin and chlorophyll metabolism | 0.001439 |
|  | SCH-N | Metabolism | Metabolism of other amino acids | D-Glutamine and D-glutamate metabolism | 0.004354 |
|  | SCH-N | Metabolism | Metabolism of other amino acids | Glutathione metabolism | 0.002786 |
|  | SCH-N | Metabolism | Metabolism of other amino acids | Selenocompound metabolism | 4.41E-05 |
|  | SCH-N | Metabolism | Metabolism of other amino acids | Taurine and hypotaurine metabolism | 0.001664 |
|  | SCH-N | Metabolism | Metabolism of other amino acids | Cyanoamino acid metabolism | 3.63E-05 |
|  | SCH-N | Metabolism | Metabolism of other amino acids | beta-Alanine metabolism | 8.65E-07 |
|  | SCH-N | Metabolism | Metabolism of other amino acids | D-Arginine and D-ornithine metabolism | 0.004958 |
|  | SCH-N | Metabolism | Metabolism of terpenoids and polyketides | Biosynthesis of vancomycin group antibiotics | 0.046269 |
|  | SCH-N | Metabolism | Metabolism of terpenoids and polyketides | Biosynthesis of ansamycins | 0.034522 |
|  | SCH-N | Metabolism | Metabolism of terpenoids and polyketides | Zeatin biosynthesis | 0.005636 |
|  | SCH-N | Metabolism | Metabolism of terpenoids and polyketides | Biosynthesis of siderophore group nonribosomal peptides | 0.003989 |
|  | SCH-N | Metabolism | Metabolism of terpenoids and polyketides | Polyketide sugar unit biosynthesis | 0.000119 |
| HC *vs.* SCH-N | SCH-N | Metabolism | Metabolism of terpenoids and polyketides | Terpenoid backbone biosynthesis | 1.52E-05 |
|  | SCH-N | Metabolism | Metabolism of terpenoids and polyketides | Carotenoid biosynthesis | 0.029158 |
|  | SCH-N | Metabolism | Metabolism of terpenoids and polyketides | Geraniol degradation | 0.000418 |
|  | SCH-N | Metabolism | Metabolism of terpenoids and polyketides | Limonene and pinene degradation | 7.97E-07 |
|  | SCH-N | Metabolism | Metabolism of terpenoids and polyketides | Biosynthesis of type II polyketide backbone | 0.000126 |
|  | SCH-N | Metabolism | Metabolism of terpenoids and polyketides | Sesquiterpenoid and triterpenoid biosynthesis | 1.02E-06 |
|  | SCH-N | Metabolism | Nucleotide metabolism | Pyrimidine metabolism | 0.009616 |
|  | SCH-N | Metabolism | Nucleotide metabolism | Purine metabolism | 0.003052 |
|  | SCH-N | Metabolism | Xenobiotics biodegradation and metabolism | Drug metabolism - other enzymes | 0.011258 |
|  | SCH-N | Metabolism | Xenobiotics biodegradation and metabolism | Nitrotoluene degradation | 0.043407 |
|  | SCH-N | Metabolism | Xenobiotics biodegradation and metabolism | Metabolism of xenobiotics by cytochrome P450 | 0.029895 |
|  | SCH-N | Metabolism | Xenobiotics biodegradation and metabolism | Toluene degradation | 0.002181 |
|  | SCH-N | Metabolism | Xenobiotics biodegradation and metabolism | Bisphenol degradation | 0.037095 |
|  |  |  |  |  |  |
| HC *vs.* SCH-N | SCH-N | Metabolism | Xenobiotics biodegradation and metabolism | Chloroalkane and chloroalkene degradation | 3.19E-05 |
|  | SCH-N | Metabolism | Xenobiotics biodegradation and metabolism | Styrene degradation | 0.000355 |
|  | SCH-N | Metabolism | Xenobiotics biodegradation and metabolism | Dioxin degradation | 0.000142 |
|  | SCH-N | Metabolism | Xenobiotics biodegradation and metabolism | Fluorobenzoate degradation | 1.74E-05 |
|  | SCH-N | Metabolism | Xenobiotics biodegradation and metabolism | Aminobenzoate degradation | 6.48E-06 |
|  | SCH-N | Metabolism | Xenobiotics biodegradation and metabolism | Caprolactam degradation | 5.69E-05 |
|  | SCH-N | Metabolism | Xenobiotics biodegradation and metabolism | Benzoate degradation | 1.65E-06 |
|  | SCH-N | Metabolism | Xenobiotics biodegradation and metabolism | Chlorocyclohexane and chlorobenzene degradation | 1.63E-05 |
|  | SCH-N | Metabolism | Xenobiotics biodegradation and metabolism | Xylene degradation | 0.010812 |
|  | SCH-N | Organismal Systems | Digestive system | Protein digestion and absorption | 0.003651 |
|  | SCH-N | Organismal Systems | Immune system | NOD-like receptor signaling pathway | 0.002211 |
|  | SCH-N | Cellular Processes | Cell growth and death | Cell cycle - Caulobacter | 0.004548 |
|  | SCH-N | Cellular Processes | Cell growth and death | Meiosis - yeast | 3.88E-05 |
|  | HC | Cellular Processes | Cellular community - prokaryotes | Biofilm formation - Vibrio cholerae | 0.024517 |
|  | SCH-N | Cellular Processes | Transport and catabolism | Peroxisome | 0.006949 |
| HC *vs.* SCH-N | SCH-N | Environmental Information Processing | Membrane transport | ABC transporters | 0.002786 |
|  | SCH-N | Environmental Information Processing | Signaling molecules and interaction | ECM-receptor interaction | 0.027069 |
|  | SCH-N | Genetic Information Processing | Folding, sorting and degradation | RNA degradation | 0.038122 |
|  | SCH-N | Genetic Information Processing | Folding, sorting and degradation | Sulfur relay system | 0.002916 |
|  | SCH-N | Genetic Information Processing | Folding, sorting and degradation | Protein export | 0.000418 |
|  | SCH-N | Genetic Information Processing | Folding, sorting and degradation | Protein processing in endoplasmic reticulum | 0.018404 |
|  | SCH-N | Genetic Information Processing | Folding, sorting and degradation | Proteasome | 0.012168 |
|  | SCH-N | Genetic Information Processing | Replication and repair | Homologous recombination | 0.047758 |
|  | SCH-N | Genetic Information Processing | Replication and repair | DNA replication | 0.009616 |
|  | SCH-N | Genetic Information Processing | Replication and repair | Mismatch repair | 0.010826 |
|  | SCH-N | Genetic Information Processing | Replication and repair | Nucleotide excision repair | 7.49E-06 |
|  | SCH-N | Genetic Information Processing | Replication and repair | Base excision repair | 8.27E-05 |
|  | SCH-N | Genetic Information Processing | Replication and repair | Non-homologous end-joining | 0.000112 |
| HC *vs.* SCH-N | SCH-N | Genetic Information Processing | Transcription | RNA polymerase | 2.79E-05 |
|  | SCH-N | Genetic Information Processing | Translation | Ribosome | 0.000919 |
|  | SCH-N | Genetic Information Processing | Translation | Aminoacyl-tRNA biosynthesis | 0.000967 |
|  | SCH-N | Human Diseases | Cardiovascular diseases | Hypertrophic cardiomyopathy (HCM) | 0.036259 |
|  | HC | Human Diseases | Infectious diseases | Vibrio cholerae infection | 0.00233 |
|  | | | | | |
| HC *vs.* SCH-P | SCH-P | Metabolism | Amino acid metabolism | Valine, leucine and isoleucine biosynthesis | 0.046947 |
|  | HC | Metabolism | Biosynthesis of other secondary metabolites | Clavulanic acid biosynthesis | 0.015669 |
|  | SCH-P | Metabolism | Biosynthesis of other secondary metabolites | Streptomycin biosynthesis | 0.009496 |
|  | SCH-P | Metabolism | Biosynthesis of other secondary metabolites | Penicillin and cephalosporin biosynthesis | 0.020897 |
|  | SCH-P | Metabolism | Biosynthesis of other secondary metabolites | Betalain biosynthesis | 0.006687 |
|  | SCH-P | Metabolism | Energy metabolism | Photosynthesis | 0.042528 |
|  | HC | Metabolism | Glycan biosynthesis and metabolism | Glycosaminoglycan degradation | 0.046947 |
|  | SCH-P | Metabolism | Glycan biosynthesis and metabolism | N-Glycan biosynthesis | 0.003351 |
|  | SCH-P | Metabolism | Lipid metabolism | Primary bile acid biosynthesis | 0.004397 |
| HC *vs.* SCH-P | SCH-P | Metabolism | Lipid metabolism | Steroid biosynthesis | 0.009208 |
|  | SCH-P | Metabolism | Metabolism of cofactors and vitamins | Pantothenate and CoA biosynthesis | 0.032979 |
|  | HC | Metabolism | Metabolism of other amino acids | Phosphonate and phosphinate metabolism | 0.00717 |
|  | SCH-P | Metabolism | Metabolism of other amino acids | beta-Alanine metabolism | 0.011394 |
|  | SCH-P | Metabolism | Metabolism of other amino acids | D-Arginine and D-ornithine metabolism | 0.008656 |
|  | SCH-P | Metabolism | Metabolism of terpenoids and polyketides | Polyketide sugar unit biosynthesis | 0.012837 |
|  | SCH-P | Metabolism | Metabolism of terpenoids and polyketides | Limonene and pinene degradation | 0.032979 |
|  | SCH-P | Metabolism | Metabolism of terpenoids and polyketides | Sesquiterpenoid and triterpenoid biosynthesis | 0.005542 |
|  | SCH-P | Metabolism | Xenobiotics biodegradation and metabolism | Chloroalkane and chloroalkene degradation | 0.049295 |
|  | SCH-P | Metabolism | Xenobiotics biodegradation and metabolism | Chlorocyclohexane and chlorobenzene degradation | 0.020324 |
|  | SCH-P | Cellular Processes | Cell growth and death | Meiosis - yeast | 0.000385 |
|  | HC | Cellular Processes | Cellular community - prokaryotes | Biofilm formation - Vibrio cholerae | 0.003238 |
|  | HC | Environmental Information Processing | Membrane transport | Phosphotransferase system (PTS) | 0.007882 |
|  |  |  |  |  |  |
| HC *vs.* SCH-P | SCH-P | Genetic Information Processing | Folding, sorting and degradation | Protein processing in endoplasmic reticulum | 0.021483 |
|  | SCH-P | Genetic Information Processing | Replication and repair | Non-homologous end-joining | 0.039447 |
|  | HC | Human Diseases | Infectious diseases | Pathogenic Escherichia coli infection | 0.016006 |
|  | HC | Human Diseases | Infectious diseases | Vibrio cholerae infection | 0.001218 |
|  | SCH-P | Human Diseases | Infectious diseases | Bacterial invasion of epithelial cells | 0.02988 |
|  | | | | | |
| BD-D *vs.* SCH-N | SCH-N | Metabolism | Amino acid metabolism | Valine, leucine and isoleucine biosynthesis | 0.0126 |
|  | SCH-N | Metabolism | Carbohydrate metabolism | C5-Branched dibasic acid metabolism | 0.016756 |
|  | SCH-N | Metabolism | Metabolism of cofactors and vitamins | Pantothenate and CoA biosynthesis | 0.018382 |
|  | SCH-N | Metabolism | Metabolism of other amino acids | D-Glutamine and D-glutamate metabolism | 0.024097 |
|  | SCH-N | Metabolism | Lipid metabolism | Fatty acid biosynthesis | 0.031262 |
|  | SCH-N | Metabolism | Glycan biosynthesis and metabolism | Peptidoglycan biosynthesis | 0.031262 |
|  | SCH-N | Metabolism | Amino acid metabolism | Phenylalanine, tyrosine and tryptophan biosynthesis | 0.031262 |
|  | SCH-N | Metabolism | Metabolism of terpenoids and polyketides | Zeatin biosynthesis | 0.032615 |
|  | SCH-N | Metabolism | Metabolism of other amino acids | Selenocompound metabolism | 0.036975 |
|  |  |  |  |  |  |
| BD-D *vs.* SCH-N | SCH-N | Metabolism | Energy metabolism | Carbon fixation pathways in prokaryotes | 0.043537 |
|  | SCH-N | Metabolism | Amino acid metabolism | Lysine biosynthesis | 0.043537 |
|  | SCH-N | Metabolism | Biosynthesis of other secondary metabolites | Streptomycin biosynthesis | 0.047164 |
|  | SCH-N | Metabolism | Metabolism of cofactors and vitamins | Lipoic acid metabolism | 0.047164 |
|  | SCH-N | Cellular Processes | Cell growth and death | Cell cycle - Caulobacter | 0.032615 |
|  | BD-D | Environmental Information Processing | Signal transduction | Wnt signaling pathway | 0.00501 |
|  | SCH-N | Genetic Information Processing | Translation | Aminoacyl-tRNA biosynthesis | 0.01455 |
|  | SCH-N | Genetic Information Processing | Translation | Ribosome | 0.015255 |
|  | SCH-N | Genetic Information Processing | Folding, sorting and degradation | Protein export | 0.017552 |
|  | SCH-N | Genetic Information Processing | Transcription | RNA polymerase | 0.04532 |
|  | | | | | |
| BD-M *vs.* SCH-P | BD-M | Metabolism | Lipid metabolism | Sphingolipid metabolism | 0.001832 |
|  | BD-M | Metabolism | Glycan biosynthesis and metabolism | Other glycan degradation | 0.001948 |
|  | BD-M | Metabolism | Glycan biosynthesis and metabolism | Glycosaminoglycan degradation | 0.005538 |
|  | BD-M | Metabolism | Carbohydrate metabolism | Galactose metabolism | 0.005849 |
|  | BD-M | Metabolism | Lipid metabolism | Glycerolipid metabolism | 0.007253 |
| BD-M *vs.* SCH-P | BD-M | Metabolism | Carbohydrate metabolism | Starch and sucrose metabolism | 0.007647 |
|  | BD-M | Metabolism | Lipid metabolism | Steroid hormone biosynthesis | 0.018735 |
|  | BD-M | Metabolism | Xenobiotics biodegradation and metabolism | Fluorobenzoate degradation | 0.020565 |
|  | BD-M | Metabolism | Biosynthesis of other secondary metabolites | Penicillin and cephalosporin biosynthesis | 0.021535 |
|  | BD-M | Metabolism | Xenobiotics biodegradation and metabolism | Toluene degradation | 0.022545 |
|  | BD-M | Metabolism | Carbohydrate metabolism | Inositol phosphate metabolism | 0.023594 |
|  | BD-M | Metabolism | Metabolism of terpenoids and polyketides | Biosynthesis of type II polyketide backbone | 0.024685 |
|  | BD-M | Metabolism | Carbohydrate metabolism | Pentose phosphate pathway | 0.029486 |
|  | BD-M | Metabolism | Metabolism of other amino acids | Cyanoamino acid metabolism | 0.029486 |
|  | BD-M | Metabolism | Amino acid metabolism | Tyrosine metabolism | 0.029486 |
|  | BD-M | Metabolism | Carbohydrate metabolism | Pentose and glucuronate interconversions | 0.030802 |
|  | BD-M | Metabolism | Carbohydrate metabolism | Fructose and mannose metabolism | 0.036573 |
|  | BD-M | Metabolism | Metabolism of terpenoids and polyketides | Terpenoid backbone biosynthesis | 0.038149 |
|  | BD-M | Metabolism | Metabolism of cofactors and vitamins | Biotin metabolism | 0.038149 |
|  | BD-M | Metabolism | Xenobiotics biodegradation and metabolism | Aminobenzoate degradation | 0.039781 |
|  | BD-M | Metabolism | Metabolism of other amino acids | Taurine and hypotaurine metabolism | 0.041471 |
| BD-M *vs.* SCH-P | BD-M | Metabolism | Carbohydrate metabolism | Ascorbate and aldarate metabolism | 0.041471 |
|  | BD-M | Metabolism | Carbohydrate metabolism | Amino sugar and nucleotide sugar metabolism | 0.04322 |
|  | BD-M | Metabolism | Xenobiotics biodegradation and metabolism | Benzoate degradation | 0.04322 |
|  | BD-M | Metabolism | Lipid metabolism | Secondary bile acid biosynthesis | 0.048838 |
|  | BD-M | Genetic Information Processing | Folding, sorting and degradation | Proteasome | 0.029486 |
|  | BD-M | Genetic Information Processing | Replication and repair | Base excision repair | 0.038149 |
|  | BD-M | Genetic Information Processing | Replication and repair | Nucleotide excision repair | 0.048838 |
|  | BD-M | Human Diseases | Infectious diseases | Epithelial cell signaling in Helicobacter pylori infection | 0.00806 |
|  | BD-M | Human Diseases | Cardiovascular diseases | Hypertrophic cardiomyopathy (HCM) | 0.049311 |
|  | BD-M | Organismal Systems | Digestive system | Protein digestion and absorption | 0.001256 |
|  | BD-M | Organismal Systems | Immune system | NOD-like receptor signaling pathway | 0.010972 |
