# Supplementary material for: Gut microbial signatures and differences in bipolar disorder and schizophrenia of emerging adulthood
Source: CNS Neurosci Ther. 2022 Dec 5;29(Suppl 1):5–17. doi: 10.1111/cns.14044 (PMC10314106; doi:10.1111/cns.14044)
Supplement: Supplementary file 9 — Table S6 [file CNS-29-5-s004.docx]

Supplementary Table 6. Comparison of clinical characteristics data and alpha diversity between the BD-M subgroup and the SCH-P subgroup

| Parameter | BD - M (n = 20) | SCH - P (n = 37) | *t / Z / χ^2^- value* | *P*-value |
| --- | --- | --- | --- | --- |
| Family history (Yes / No) ^c^ | 3/17 | 13/24 | χ^2^ = 2.607 | 0.106 |
| Total disease course (months) ^b^ | 19.50 (2.25, 46.50) | 9.00 (2.00, 13.25) | Z = -1.315 | 0.189 |
| YMRS ^b^ | 32.50 (27.00, 35.50) | 14.00 (11.00, 18.50) | Z = - 5.657 | < 0.001 |
| PANSS positive symptom score ^b^ | 19.00 (15.25, 22.75) | 25.00 (20.00, 27.25) | Z = - 4.071 | < 0.001 |
| PANSS total score ^a^ | 59.70 ± 11.68 (42.00, 82.00) | 84.90 ± 12.12 (66.00, 110.00) | t = - 10.081 | < 0.001 |
| GAF ^b^ | 45.00 (43.25, 49.00) | 35.00 (31.25, 36.25) | Z = - 5.183 | < 0.001 |
| Sobs ^a^ | 169.15 ± 51.74 (95.00, 265.00) | 176.22 ± 59.16 (85.00, 351.00) | *t* = -0.449 | 0.655 |
| Ace ^b^ | 203.65 (169.15, 275.17) | 202.61 (168.37, 249.68) | *Z* = -0.084 | 0.933 |
| Chao1 ^b^ | 212.01 (165.16, 291.69) | 210.56 (166.59, 242.95) | *Z* = -0.150 | 0.880 |
| Shannon ^a^ | 4.20 ± 0.77 (1.92, 5.17) | 4.13 ± 0.91(1.85, 5.72) | *t* = 0.231 | 0.818 |
| Simpson ^b^ | 0.90 (0.86, 0.93) | 0.90 (0.80, 0.93) | *Z* = -0.368 | 0.713 |
| PD-whole tree ^b^ | 20.87 (17.78, 26.93) | 22.35 (19.10, 25.59) | *Z* = -0.903 | 0.367 |

Abbreviations: ^a^ Student's t-test; ^b^ Mann-Whitney U; ^c^ Chi-square analysis; Sob, Observer-species
