# Supplementary material for: Gut microbial signatures and differences in bipolar disorder and schizophrenia of emerging adulthood
Source: CNS Neurosci Ther. 2022 Dec 5;29(Suppl 1):5–17. doi: 10.1111/cns.14044 (PMC10314106; doi:10.1111/cns.14044)
Supplement: Supplementary file 10 — Table S7 [file CNS-29-5-s006.docx]

Supplementary Table 7. Selected genera markers by random forest models

| Genus ID | Enrichment | |
| --- | --- | --- |
| **HC *vs.* BD** | | |
| Escherichia-Shigella | | BD |
| Ruminococcus | | HC |
| Coprococcus | | HC |
| Prevotellaceae_NK3B31_group | | BD |
| norank_f__norank_o__Clostridia_UCG-014 | | HC |
| Collinsella | | HC |
| Hungatella | | BD |
| Subdoligranulum | | HC |
| Parasutterella | | BD |
| Dorea | | HC |
| Monoglobus | | HC |
| Allorhizobium-Neorhizobium-Pararhizobium-Rhizobium | | HC |
| Bilophila | | BD |
| Agathobacter | | HC |
| Flavonifractor | | BD |
| Megamonas | | HC |
| Dialister | | HC |
| Fenollaria | | BD |
| Odoribacter | | BD |
| Candidatus_Soleaferrea | | BD |
| Fournierella | | BD |
| Sellimonas | | HC |
| Lachnoclostridium | | BD |
| Oribacterium | | BD |
| Pseudomonas | | BD |
| unclassified_f__Oscillospiraceae | | BD |
| Butyricimonas | | BD |
| norank_f__Coriobacteriales_Incertae_Sedis | | HC |
| Alkanindiges | | HC |
| Eubacterium_ruminantium_group | | HC |
| Eubacterium_siraeum_group | | HC |
| Lachnospiraceae_UCG-006 | | BD |
| GCA-900066755 | | BD |
| Colidextribacter | | BD |
| Actinomyces | | HC |
| Anaerofilum | | BD |
| unclassified_k__norank_d__Bacteria | | BD |
| Ruminococcus_gnavus_group | | BD |
|  | |  |
| **HC *vs.* SCH** | | |
| Monoglobus | | HC |
| Prevotellaceae_NK3B31_group | | SCH |
| Escherichia-Shigella | | SCH |
| Subdoligranulum | | HC |
| Coprococcus | | HC |
| Ruminococcus | | HC |
| Turicibacter | | SCH |
| Butyricicoccus | | HC |
| Faecalibacterium | | HC |
| unclassified_k__norank_d__Bacteria | | SCH |
| Alcaligenes | | HC |
| Ruminococcus_torques_group | | HC |
| Eubacterium_ruminantium_group | | HC |
| Allorhizobium-Neorhizobium-Pararhizobium-Rhizobium | | HC |
| Erysipelotrichaceae_UCG-003 | | HC |
| Blautia | | HC |
| Lachnospiraceae_NK4A136_group | | HC |
| Adlercreutzia | | HC |
| unclassified_f__Lachnospiraceae | | HC |
| Eubacterium_siraeum_group | | SCH |
| Prevotellaceae_UCG-001 | | SCH |
| Lachnoclostridium | | SCH |
| Fusicatenibacter | | SCH |
| Dorea | | HC |
| norank_f__norank_o__Clostridia_UCG-014 | | HC |
| Haemophilus | | HC |
| Enterobacter | | SCH |
| Holdemania | | SCH |
| Eubacterium_nodatum_group | | SCH |
| Klebsiella | | SCH |
| Megasphaera | | HC |
| GCA-900066575 | | SCH |
|  | |  |
| **BD *vs.* SCH** | | |
| Bilophila | | BD |
| Megamonas | | SCH |
| Parasutterella | | BD |
| Alcaligenes | | BD |
| Eggerthella | | SCH |
| Cloacibacillus | | BD |
| Psychrobacter | | BD |
| Lachnospiraceae_UCG-010 | | BD |
| Faecalibacterium | | BD |
| Anaerofilum | | BD |
| Akkermansia | | BD |
| Acetanaerobacterium | | BD |
| Ezakiella | | BD |
|  | |  |
| **BD-D *vs.* SCH-N** | | |
| Odoribacter | | SCH-N |
| Desulfovibrio | | SCH-N |
| Bilophila | | BD-D |
| CAG-352 | | BD-D |
| Lachnospiraceae_UCG-010 | | BD-D |
| Weissella | | BD-D |
| Lactobacillus | | SCH-N |
| Christensenellaceae_R-7_group | | BD-D |
| Ezakiella | | BD-D |
| Romboutsia | | SCH-N |
| Prevotellaceae_NK3B31_group | | SCH-N |
| Campylobacter | | BD-D |
| Butyricimonas | | BD-D |
| Lachnospiraceae_UCG-004 | | SCH-N |
| unclassified_f_Ruminococcaceae | | BD-D |
| Faecalibacterium | | BD-D |
|  | |  |
| **BD-M *vs.* SCH-P** | | |
| Flavonifractor | | BD-M |
| Haemophilus | | SCH-P |
| Parasutterella | | BD-M |
| unclassified_o_Oscillospirales | | SCH-P |
| Coprobacter | | SCH-P |
| UCG-009 | | SCH-P |
| Coprococcus | | SCH-P |
| norank_f_Christensenellaceae | | SCH-P |
| Faecalibacterium | | BD-M |
| Megamonas | | SCH-P |
| Escherichia-Shigella | | BD-M |
| Weissella | | SCH-P |
| Enhydrobacter | | BD-M |
| Eubacterium_ventriosum_group | | SCH-P |
| Bilophila | | BD-M |
| Brevundimonas | | SCH-P |
| Collinsella | | SCH-P |
| Lachnospiraceae_UCG-004 | | BD-M |
| Clostridium_innocuum_group | | BD-M |
| Family_XIII_AD3011_group | | SCH-P |
| Lachnospiraceae_NK4A136_group | | SCH-P |
| Granulicatella | | SCH-P |
| Allisonella | | SCH-P |
| Alcaligenes | | BD-M |
| norank_f__norank_o__Rhodospirillales | | SCH-P |
| Hydrogenoanaerobacterium | | SCH-P |
| Delftia | | SCH-P |
| Methylobacterium-Methylorubrum | | BD-M |
| Rikenellaceae_RC9_gut_group | | BD-M |
| NK4A214_group | | SCH-P |
| CAG-352 | | BD-M |
